# Tension Monitoring during Epithelial-to-Mesenchymal Transition Links the Switch of Phenotype to Expression of Moesin and Cadherins in NMuMG Cells

**DOI:** 10.1371/journal.pone.0080068

**Published:** 2013-12-05

**Authors:** David Schneider, Thilo Baronsky, Anna Pietuch, Jan Rother, Marieelen Oelkers, Dagmar Fichtner, Doris Wedlich, Andreas Janshoff

**Affiliations:** 1 Institute of Physical Chemistry, Georg-August-University Göttingen, Göttingen, Germany; 2 Institute for Cell and Developmental Biology, Karlsruhe Institute of Technology (KIT), Fritz Haber Weg 2, Karlsruhe, Germany; University of Zurich, Switzerland

## Abstract

Structural alterations during epithelial-to-mesenchymal transition (EMT) pose a substantial challenge to the mechanical response of cells and are supposed to be key parameters for an increased malignancy during metastasis. Herein, we report that during EMT, apical tension of the epithelial cell line NMuMG is controlled by cell-cell contacts and the architecture of the underlying actin structures reflecting the mechanistic interplay between cellular structure and mechanics. Using force spectroscopy we find that tension in NMuMG cells slightly increases 24 h after EMT induction, whereas upon reaching the final *mesenchymal-like* state characterized by a complete loss of intercellular junctions and a concerted down-regulation of the adherens junction protein E-cadherin, the overall tension becomes similar to that of solitary adherent cells and fibroblasts. Interestingly, the contribution of the actin cytoskeleton on apical tension increases significantly upon EMT induction, most likely due to the formation of stable and highly contractile stress fibers which dominate the elastic properties of the cells after the transition. The structural alterations lead to the formation of single, highly motile cells rendering apical tension a good indicator for the cellular state during phenotype switching. In summary, our study paves the way towards a more profound understanding of cellular mechanics governing fundamental morphological programs such as the EMT.

## Introduction

The selective transition from the epithelial to the mesenchymal cellular phenotype is an essential process during morphogenesis [1]. The epithelial-to-mesenchymal transition (EMT) encompasses biological processes such as dispersion of cells in embryos, wound healing, and initiating the invasive and metastatic behavior of epithelial cancers [2], [3], [4]. Although much is known about the molecular cues that are responsible for EMT [5], [6], the interplay between structure, dynamics and mechanical response is only poorly understood so far [7], [8]. The ability of mesenchymal cells to migrate, originates from a huge set of structural, mechanical and dynamic alterations during EMT, which are triggered by extracellular signals and intracellular transcription factors [9], [10]. These substantial structural changes pose a considerable challenge for the formerly polar cell to maintain the plasma membrane's integrity. Considering that area dilatation of the plasma membrane is limited to merely 3–5% of its initial area until lysis occurs, severe shape changes need to be balanced by careful adjustment of membrane tension through regulation of the available surface area commonly referred to as membrane tension homeostasis [11].

The mechanical behavior of cells is mainly governed by an intricate interplay between membrane mechanics and the associated cytoskeleton consisting of actin, myosin and intermediate filaments [12]. Particularly, the actomyosin cortex is responsible for the regulation of cellular mechanics and cellular shape due to its highly organized network-like structure and its capability of actively generating forces using motor proteins [13]. Albeit the cytoskeleton is indisputably essential for the mechanical response, evidence accumulates that the actomyosin cortex generates lateral tension in the plasma membrane to resist mechanical stimuli as a first order effect [14]. Apical tension is determined and influenced by a number of processes comprising osmotic pressure, coupling strength of the actin cytoskeleton to the membrane via ezrin-radixin-moesin proteins (ERM proteins), actomyosin contractility, as well as tension induced via cell-cell contacts and cell-ECM adhesion sites [11], [15], [16]. In this context, invaginations such as caveolae as well as protrusions like microvilli are known to buffer changes in tension by sacrificing membrane material [17], [18]. This is the reason why homeostasis of tension achieved by regulation of surface area is pivotal to compensate external and internal stress that might lead to lysis of the plasma membrane. Hence, the question arises how cells that undergo the EMT sense and adjust lateral tension to prevent lysis of the plasma membrane during the switch in phenotype.

Here, we investigate the simultaneous changes in mechanics *and* cellular structure of the epithelial cell line NMuMG during TGF-β1 induced epithelial-to-mesenchymal transition [19] with emphasis on spatiotemporal alterations in apical tension comprising lateral tension within the membrane itself and cortical tension mediated via the actin cytoskeleton, as well as concomitant changes in surface area. We found that during EMT apical tension increases with exposure time to TGF-β1, while excess surface area of the apical membrane decreases visibly. This mechanical transition therefore generates substantially stiffer cells compared to ordinary polar epithelial cells. Although storage of excess membrane is almost exhausted during EMT, the calculated tension never exceeds 2 mN/m, which is well below lysis tension (10 mN/m) [20]. Eventually, the mechanical properties of individual mesenchymal cells approach the elastic signature of fibroblasts [21]. The influence of the actin cytoskeleton and its structure on apical tension increases significantly during EMT, which is reflected in a deviation of the detected tension values from indentation experiments in comparison to those obtained from tether pulling experiments. This can be explained by rearrangement of the cortical actin in support of stress fiber formation and the concomitant redistribution and clustering of ERM proteins like moesin at the plasma membrane, which is supposed to be a prerequisite for EMT [22]. Changes in the cytoskeletal structure also provoke loss of cell-cell contacts characterized by depletion of E-cadherin. Employing single molecule and single-cell force spectroscopy we found that the characteristic *trans*-interaction of E-cadherin molecules prior to EMT is replaced by a significant weaker interaction with lower forces, which we attribute to destabilization of the adherence junction complex and delocalization of E-cadherin within the membrane. In summary, we were able to determine intercellular junctions, surface area as well as the cytoskeletal architecture as main regulators for tension during epithelial-to-mesenchymal transition.

## Materials and Methods

### Cell culture and reagents

The mouse mammary epithelial cell line NMuMG (ATCC, Manassas, VA), was maintained in Dulbecco's modified Eagles medium with 4.5 g/l glucose (Lonza, Köln, Germany), 10% fetal calf serum (PAA Laboratories GmbH, Cölbe, Germany) and 4 mM L-glutamine (Biochrom, Berlin, Germany). Additionally, 10 µg/ml insulin (Sigma-Aldrich, Munich, Germany) was added as stated in the cultivation protocol by ATCC (American Type Culture Collection). During all measurements, the regular culture medium supplemented with 15 mM Hepes, Amphotericin and Penicillin/Streptomycin (all PAA Laboratories GmbH) was used. For all of the measurements mentioned below NMuMG cells were seeded to 70% confluency on Petri dishes without any substrate coating providing sufficient area for morphological changes during EMT. Fibroblasts obtained from 2-d-old rats were kindly provided by Marco Tarantola, Katharina Schneider and Marion Kunze (Max Planck Institute for Dynamics and Self-Organization, Göttingen). During the measurement fibroblasts were kept in Dulbecco's modified Eagles medium (Invitrogen, Karlsruhe, Germany) supplemented with 1% L-glutamine (Invitrogen), 10% FCS (Invitrogen) and 1% penicillin/streptomycin (Life Technologies). All preparations were done in regard to the guidelines of the German Animal Protection Act (TierSchG) and were reported to the responsible animal welfare representative. All animals were killed before preparations by decapitation, which conforms with the ‘Guidelines for Euthanasia of Rodent Fetuses and Neonates’ of the NIH, and were reported referred to §4 para. 3 TierSchG. Since animal testing was not conducted the protocol did not require approval by the Committee on the Ethics of Animal Experiments. Necessary certificates of exemption for technical staff members were obtained from the Lower Saxony State Office for Consumer Protection and Food Safety (Laves) with respect to §9 para. 1 sentence 4 (TierSchG). Tissue was only used to prepare cultures of cardiomyocytes and fibroblasts. TGF-β1 was obtained from Life Technologies GmbH (Darmstadt, Germany) and used in a final concentration of 10 ng/mL diluted in culture medium including Hepes, Amphotericin and Penicillin/Streptomycin. EDTA was received from Sigma-Aldrich and used in a final concentration of 2 mM diluted in culture medium with Hepes, Amphotericin and Penicillin/Streptomycin.

Cytochalasin D was obtained from Life Technologies and used in a final concentration of 10 µg/mL diluted in culture medium including Hepes, Amphotericin and Penicillin/Streptomycin. For single molecule experiments control measurements an E-cadherin antibody (Life Technologies GmbH) diluted in serum-free medium to a final concentration of 200 µg/mL was employed. Prior to measuring, cells were incubated with the antibody for 1 h. Thereafter, the serum-free medium was removed and fresh culture medium supplemented with 15 mM Hepes was added. The inhibitors rapamycin, cycloheximide, blebbistation and Y-27632 were purchased from Sigma-Aldrich and used in concentrations as indicated. Generally, application of rapamycin leads to a decline in both cell motility and *de novo* protein synthesis via inhibition of the mTOR signaling pathway in TGF-β1-stimulated NMuMG cells [23]. Cycloheximide is a well-known inhibitor for protein synthesis by blocking translational elongation [24]. Blebbistatin prevents rebinding of the myosin head to the actin cytoskeleton, thereby decreasing acto-myosin contractility within treated cells [25]. Finally, Y-27632 is a Rho kinase inhibitor interfering with the formation of actin stress fibers [26]. The impact of these compounds on cellular dynamics and presence of cell-cell contacts during EMT of NMuMG cells has recently been investigated by us in a separate study [27].

### Fluorescence microscopy

Immunostaining combined with fluorescence microscopy was carried out using an upright microscope (Olympus BX51, 20× or 40× water immersion). Cells were grown on Ibidi™ petri dishes (Ibidi, Martinsried, Germany), treated with 10 ng/ml of TGF-β1 for either 48 h or 10 d and compared to untreated reference samples. Fixation of the cells was carried out using a 4% paraformaldehyde solution (Sigma-Aldrich), and an incubation time of 15 min. This was followed by washing the cells twice with 1× PBS (PAA) without Ca^2+^ and Mg^2+^ denoted PBS^−^ throughout the manuscript and permeabelizing them with a 1× PBS^−^ solution containing 0.4% Triton-X (Sigma-Aldrich) and 5% BSA (Sigma-Aldrich) to block unspecific binding sites. For E-cadherin staining cells were incubated for 1 h with a monoclonal Alexa488-conjugated IgG2a antibody (BD Bioscience) diluted to a final concentration of 5 µg/mL in 1× PBS^−^ supplemented with 1% BSA and 0.3% Triton-X. For DNA staining 4′-6-diamidino-2-phenylindole (DAPI, Sigma-Aldrich) was used and an incubation time of 15 min was chosen.

### Indentation experiments with conical indenter

Force indentation curves on cell layers were recorded using a Nanowizard II setup (JPK Instruments, Berlin, Germany) placed on an inverted Olympus IX81 microscope (Hamburg, Germany). Silicon nitride cantilevers (MLCT, Bruker Daltonics, Camarillo, USA) with a nominal spring constant of 0.01 N/m were employed. A petri dish heater (JPK Instruments) set to 37°C as well as Hepes-buffered culture medium supplemented with Amphotericin and Penicillin/Streptomycin were used to assure physiological conditions throughout all experiments.

During indentation experiments, the indentation force of either 500 or 1000 pN, and the velocity of cantilever approach *and* retraction (1 µm/s) were kept constant. Analysis of the recorded force-distance curves was conducted using a tension based model considering the properties of adhering cells in contrast to non-adhering cells according to the following equations:
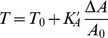
(1)

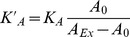
(2)The complete theory is described precisely in literature [28], [29], [30] and a detailed derivation given in the supporting information (Supplementary Text S1). In brief, the force *T* acting against cantilever indentation during deformation of the cellular structures strongly depends on indentation depth. Whereas apical tension *T_0_* arising from membrane tension *and* cortical tension dominates the elastic response at lower values, stretching of the cellular membrane expressed in the area compressibility modulus *K_A_* occurs at higher indentation depth. The influence of both parameters on the shape of the recorded force-distance curves is visualized in Fig. S1 and Fig. S2. Certainly stretching of the membrane during indentation strongly depends on the amount of free accessible membrane material. Therefore, *ΔA* and *A_0_* describe the area change due to indentation of the conical indenter and the apical side of the spread cell excluding folds or invaginations, respectively. Due to the fact that the excess area of cellular membranes (*A_Ex_*), bearing mainly membrane folds and invaginations, is unknown, only an apparent area compressibility modulus *K_A_^′^* can be determined (equation 2).

**Figure 1:**
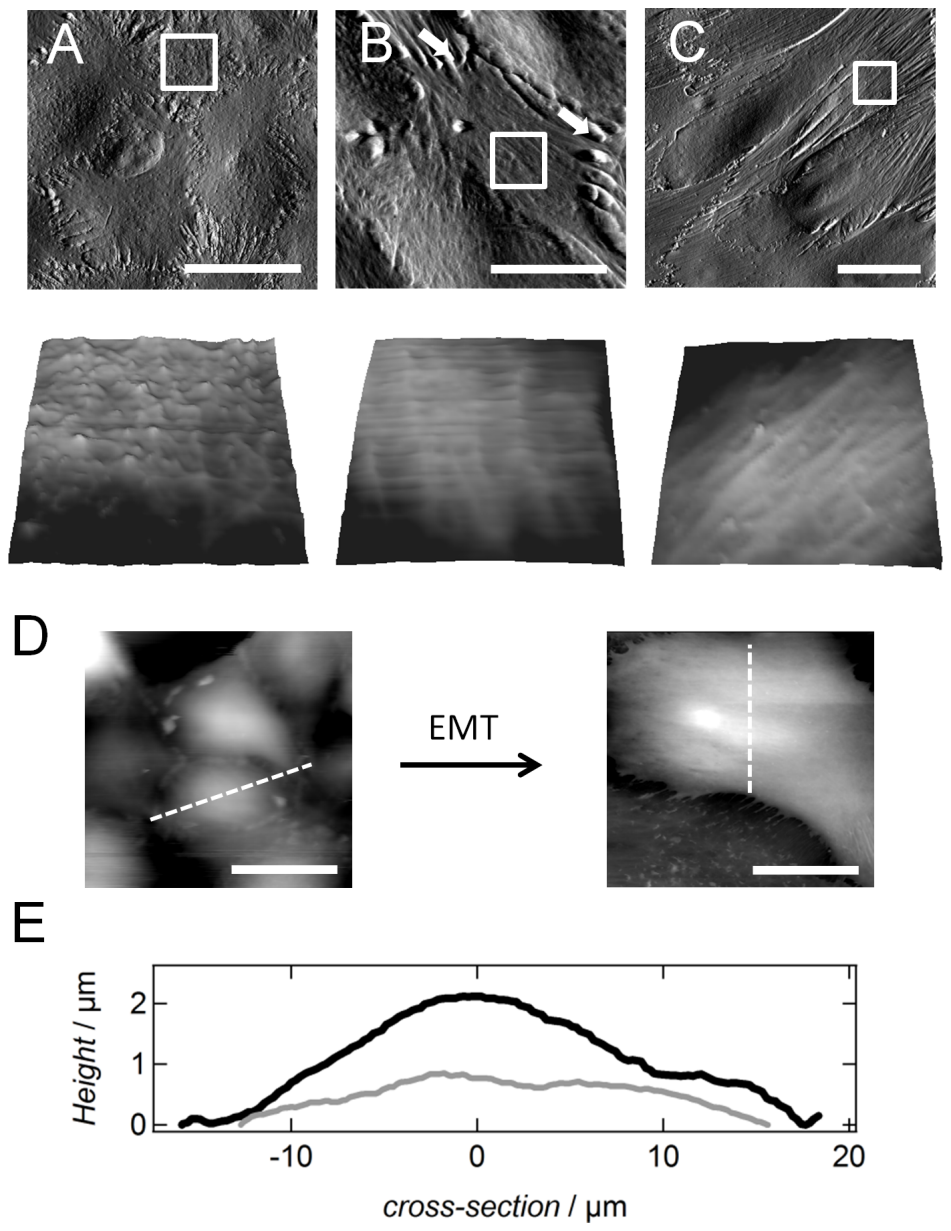
Structural alterations during epithelial-to-mesenchymal transition. (A–C) AFM deflection images of fixed untreated and fixed TGF-β1 treated NMuMG cells (24 h or 48 h incubation time, B and C, respectively). Fixation was carried out using glutardialdehyde (10 min incubation time) leading to cross-linking of proteins. Subsequent loss of cell-cell junctions after 24 h of incubation is marked by white arrows. Zoom-ins of the corresponding AFM height images show a loss of cellular protrusions during EMT. White boxes in the deflection images mark the chosen regions for the zooms. Size of the height images is 9.3×9.3 µm^2^. (D) AFM height images of living untreated and living treated (48 h incubation time) NMuMG cells. (E) The obtained cross-sections from these images provide information about the contact angle, the radius and the height of the cells before (black) and after the transition (grey). All AFM images were recorded in closed-loop contact mode using MLCT cantilevers and a scan rate of 0.2 Hz. Scale bars: 20 µm.

Within this study, we focused on pre-tension *T_0_* as this parameter is directly accessible from the recorded force curves even without knowledge of the buried membrane area. Bending and shearing of the membrane are neglected. According to our model, cells are treated as spherical caps with characteristic radii and contact angles for each cell type before and after the transition. This information was obtained from AFM images (Fig. 1 D/E). The images were recorded using a closed loop feedback circuit and the piezo movement was corrected with the help of capacitive sensors implemented into the scanner of the AFM (JPK instruments). The mean relative height and mean radius were calculated by analyzing at least 3 cells per category. For NMuMG cells treated with either EDTA or cytochalasin D and NMuMG cells before the transition, a mean radius of 10.1 µm and a mean relative height of 2.2 µm for the spherical cap are computed. After 24 h of TGF-β1 incubation we found a radius of 10.3 µm and a relative height of 1.8 µm, whereas upon reaching the final *mesenchymal-like state* a radius of 10.3 µm and a relative height of 1.4 µm for the cells were used as input parameters for the indentation model. Similar values were used for *mesenchymal-like* NMuMG cells incubated with rapamycin, cycloheximide, blebbistatin, Y-27632 or cytochalasin D. Finally, for single untreated NMuMG cells parameters of 12.3 µm and 2.0 µm are determined. Adapting the input parameters in dependence of the cellular state during EMT is necessary as the morphological changes influence the elastic response upon cantilever indentation as inferred from simulation studies (Fig. S3 and S4). Although we performed indentation experiments with pyramidal shaped MLCT cantilevers, we used a conical shape as input parameter for tension model. Due to the asymmetry of the MLCT cantilevers a calculation of the cellular deformation would not be tractable analytically. However, considering a conformal contact our model is valid as the contact area of both shapes differs only by a factor of 1.3 comparing a conical indenter with a pyramidal one. For calculation an mean opening angle of 17.5 was assumed for the conical/pyramidal tip.

### Indentation experiments with colloidal probe

A similar setup was used here as for the indentation experiments with a conical indenter (*vide supra*). Spherical glass beads (Duke borosilicate glass 9015, Duke Scientific Corporation, Palo Alto, CA, USA) with a diameter of 15 µm were glued onto tip-less silicon nitride cantilevers for indentation experiments (tipless MLCT-O10, Bruker Daltonics) using an epoxy resin cured at a temperature of about 90°C (Epikote 1004, Brenntag GmbH, Mühlheim, Germany). The bead was placed onto the cantilever with the help of a nanomanipulator (MM3A-LS, Kleindiek Nanotechnik GmbH, Reutlingen, Germany). Cantilever retraction and approach were kept constant at 1 µm/s. Cells were indented in the center.

In order to extract apical tension *T*_0_ of NMuMG cells in the absence and presence of TGF-β1 from colloidal probe force measurements, we employed a theoretical approach devised by Lomakina and coworkers [31]. Essentially, the approach is based on determining the pressure change *P* of a bead (radius *R*_b_), the colloidal probe, in conformal contact with the cell of radius *R_c_* using Laplace's law:
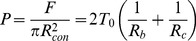
(3)providing the force *F* acting on the cell. The contact radius *R_con_* can be used to compute the indentation depth *z*:
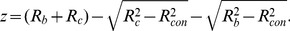
(4)With equations (3) and (4) the force-indentation curve can be modeled to estimate tension values from colloidal probe measurements.

### Tether pulling experiments

For tether pulling experiments the same setup as for indentation experiments with pyramidal indenters was used. Tips of MLCT cantilevers were first cleaned with argon plasma for 1 min (Plasma Cleaner, Harrick, NY, USA) followed by rinsing with isopropanol (VWR International GmbH, Darmstadt, Germany). Finally, the cantilevers were incubated with concanavalin A (Sigma-Aldrich) diluted in 1× PBS^−^ to a final concentration of 2.5 mg/mL for 30 min at room temperature. Concanavalin A is known for strong binding to carbohydrates on the cellular surface leading to tether formation upon cantilever retraction [32]. Measurements were performed in Hepes-buffered cell medium with Amphotericin and Penicillin/Streptomycin. We chose an approach/retraction velocity of 1 µm/s, a setpoint of 500 pN and a contact time of 1 s between the tip and the cells. Using the following equation, membrane tension from tether pulling experiments before and after the transition is computed [33].
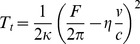
(5)Besides static membrane tension *T_t_* membrane viscosity *η* has to be taken into consideration. *v* denotes the velocity of tether pulling. The bending modulus of the cellular membrane was assumed to be *κ* = 2.7e^−19^ as obtained by Dai and Sheetz (1999) for renal epithelial cells [34]. *C* is a correction factor with a value of 1.6 [35].

### Single-cell force spectroscopy (SCFS)

Measurements were accomplished using a Cellhesion200 setup (JPK Instruments) combined with an Olympus IX81 microscope. Again we assured physiological conditions using a Petri dish heater (JPK Instruments) set to 37°C. Tipless cantilevers (Arrow TL-2, Bruker Daltonics) with a nominal spring constant of 0.03 N/m were employed for the experiment and, prior cell picking, they were washed twice in isopropanol and ultra-pure water. Additionally, cantilevers were sterilized using argon plasma for 1 min. The cleaned cantilevers were then incubated for 15 min in a 30 mM CellTak solution (BD Biosciences) diluted in 1× PBS^−^ for 15 min at room temperature. NMuMG cells were seeded onto Petri dishes in Hepes-buffered cell culture medium at least 48 h before starting the measurement ensuring formation of proper cell-substrate and cell-cell adhesion sites. In case of the *mesenchymal-like* cells, incubation times of at least 8 d in presence of the cytokine TGFβ-1 were applied to guarantee a complete EMT and down-regulation of E-cadherin. Thereafter, freshly suspended cells, either epithelial ones or *mesenchymal-like* ones, were added to the Petri dish and a single cell was captured using the Celltak-coated cantilever. Using the cell as a probe, it is brought into contact with either a spread epithelial or *mesenchymal-like* cell. A contact force of 500 pN and an approach/retraction velocity of 10 µm/s were employed for all measurements. Furthermore, we chose a contact time of 1 s between the cells in either state since at longer times of up to 10 s the variances of detachment forces increase considerably, which in turn reduces significance of the obtained results (*data not shown*). All adhesion measurements were again conducted in cell medium supplemented with Hepes, Amphotericin and Penicillin/Streptomycin.

### Single-molecule force spectroscopy (SMFS)

SMFS was carried out with a Nanowizard II setup (JPK Instruments) mounted on an inverted Olympus IX81 microscope. We used gold-coated cantilevers (Bio-lever, BL-RC150VB, Olympus, Japan) with a nominal spring constant of 0.06 N/m. First, cantilevers were cleaned in argon plasma for 30 s and subsequently functionalized by incubation for 3 h in a 1 mM benzylguaninthiol : matrix-thiol (MeO) (1∶100) solution dissolved in isopropanol. This was followed by rinsing the cantilevers with pure solvent and finally with buffer (10 mM HEPES, 100 mM NaCl, 1 mM EDTA). Thereafter, cantilevers were incubated with protein solution (2 µM) to couple Ecad15 (E-cadherin including all 5 ectodomains) to the tip. This coupling is induced via a Snap-Tag C-terminally fused to Ecad15. The coupling procedure is described in detail by Wedlich and coworkers [36]. Force-distance measurements were performed using a pulling speed of 1 µm/s and a contact force of 100 pN. The contact time between the functionalized cantilever and either the epithelial or *mesenchymal-like* cell was kept constant (1 s). The matrix-thiol, Methoxy-capped tri(ethyleneglycol)undecanthiol, was synthesized at the Fraunhofer Institute for Manufacturing Technology and Applied Materials Research (IFAM). Benzylguanine-terminated disulfide (BGT) was obtained from New England Biolabs (Ipswich, USA). The E-cadherin construct is expressed in stably transfected HEK293 cells and purified afterwards.

### AFM Imaging and roughness analysis

AFM imaging of NMuMG cells was accomplished using a Nanowizard II setup. Due to lateral mobility of the cellular membrane, cells were fixed using a 2.5% glutardialdehyd solution (Fisher Scientific GmbH, Nidderau, Germany) diluted in 1× PBS^−^ (15 min incubation time) prior to scanning. MLCT cantilevers with a nominal spring constant of 0.01 N/m were employed. A scan rate of 0.2 Hz was used for contact-mode imaging.

Roughness of the cells within the different stages during EMT was analyzed as follows. In a first step, a user-defined area of 9.7×9.7 µm^2^ on any cellular body was chosen for analysis. Subsequently, a general trend was subtracted from this section to correct for differences in cellular height. Therefore a 2-D sliding window of 11×11 points was adopted. In order to avoid boundary problems when applying the moving average box, the images were mirrored in both x and y direction. The algorithm computes variances for each row perpendicular to the scan direction of the AFM cantilever. Finally, the mean variances for each category were computed. In case of NMuMG cells within the epithelial state as well as for NMuMG cells from the *mesenchymal-like* state three different areas were chosen for analysis, whereas for the transitional state one area was selected (Fig. S5).

### AFM rheology measurements and data acquisition

A MFP-3D atomic force microscope (Asylum Research, Santa Barbara, CA, USA) placed on an inverted IX51 Olympus microscope was used for rheological measurements. The cantilever (MLCT, Bruker Daltonics) was excited to a sinusoidal oscillation (5 Hz to 200 Hz) at a defined indentation depth *δ_0_* within the samples. According to a model introduced by Alcaraz and coworkers [37], we were able to obtain quantitative information about the elastic and viscous properties of our samples from the recorded datasets. Although Hertzian contact mechanics forms the base, the model has to be extended by an additional frequency-independent tension term as already described [38]. Considering a correction for the hydrodynamic drag [39] and a linearization for small amplitude oscillations [21], the complex shear modulus G*, as well as the storage *G′* and loss modulus *G″* were obtained. According to the Hertz model only force-distance curves with low indentation depths of 50–550 nm were analyzed in order to avoid influence of the underlying substrate or stiff intracellular structures like the nucleus and to stay in the limits of the model [40]. Similarly to the indentation and adhesion measurements cells were investigated under physiological conditions using Hepes-buffered medium and a Petri dish heater (JPK Instruments) adjusted to 37°C.

### Cantilever calibration

Spring constants of all cantilevers were determined after cleaning (Ar plasma, 1 min) but before the individual incubation steps using the thermal noise method [41]. Although cells are mostly grown to confluent monolayers, we always found uncovered spots on the Petri dishes to calibrate the cantilever. All of the force measurements mentioned above are then performed on cells which are several mm away from the free area.

### Statistics

To evaluate the statistics of the obtained data, a non-parametric statistical hypothesis test, Wilcoxon rank-sum test, was employed as indicated accounting for non-normal data. The alpha level was either set to 0.1 or 0.01.

## Results

### Structural alterations of NMuMG cells during EMT

Figure 1 shows AFM deflection, as well as relative height images of untreated (Fig. 1A) and treated NMuMG cells incubated for either 24 h (Fig. 1B) or 48 h (Fig. 1C) with the cytokine TGF-β1. During EMT, we observe a dramatic change of the cellular shape including an increased formation of stress fibers and a loss of cell-cell contacts. Furthermore, a decrease in the number of membrane protrusions, as well as a decline in the cellular height of the cellular cap during EMT (Fig. 1D,E) are clearly visible in the recorded AFM images underscoring the considerable structural alterations during the transformation. The surface structure of epithelial and *mesenchymal-like* cells shows huge differences in corrugation supporting the idea of severe changes in membrane area and structure during transition. Whereas in the epithelial state membrane folds and protrusions are numerous, the surface during EMT adopts a much smoother topography already after 24 h of incubation with TGF-β1. To quantify these observations, we performed a roughness analysis of the AFM height images as described in the Materials and Methods section (Fig. S5). By doing so, we could unequivocally show that the calculated variances of the cellular surface heights decrease significantly from 480 nm^2^±190 nm^2^ (*n* = 300) within the epithelial state to 370 nm^2^±110 nm^2^ (*n* = 71, p-value<0.01, Wilcoxon rank sum test) during the transitional state down to 350 nm^2^±180 nm^2^ (*n* = 209, p-value<0.01, Wilcoxon rank sum test) after completion of EMT. However, due to the decline in cellular height and the concomitant rearrangement of the actin cytoskeleton during the transition, stress fibers become visible upon AFM imaging. These putative corrugations are also captured by our roughness analysis leading to a decline in significance of the calculated variances. Consequently, the amount of free accessible membrane material allocated by membrane protrusions is even smaller than expected from roughness analysis, particularly in the latter stages of development. However, these observations are in good agreement with results from literature describing a strong influence of TGF-β1 on this epithelial cell line [42].

### Alterations of cellular mechanics during EMT

**Figure 2:**
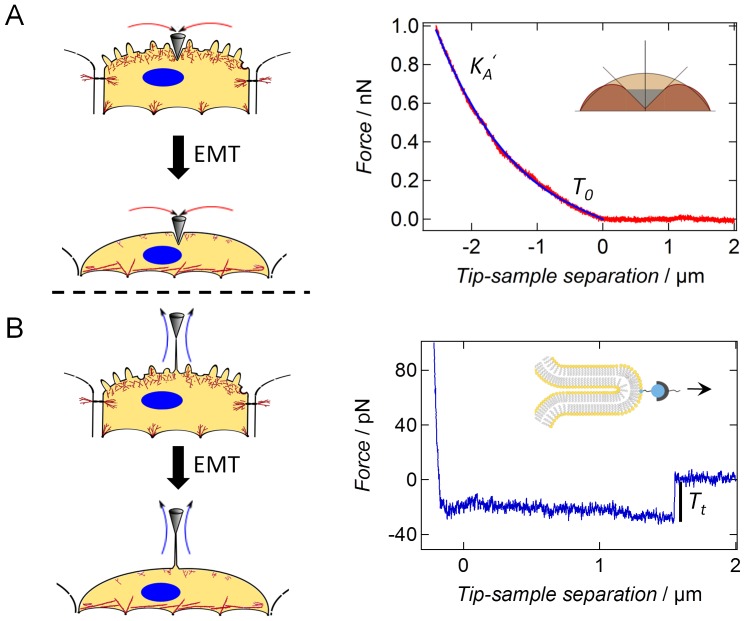
Scheme showing (A) AFM indentation and (B) tether pulling experiments before and after the epithelial-to-mesenchymal transition (EMT). The dashed arrows indicate the direction of force exerted to the membrane cortex upon either indentation or pulling. (A) Force indentation curves are analyzed using a tension model model [28], [30], [43]. Exemplarily chosen force-distance curve (red) and the corresponding fit by applying the extended indentation model (blue). Whereas *T_0_* dominates the elastic response upon low indentation depths, the influence of *K_A_* increases at higher indentations a lateral stretching of the cellular shell occurs. A typical AFM force-distance curve of an untreated NMuMG cell including cantilever approach is shown (red). (B) During cantilever retraction (blue) the characteristic formation of a tether at constant force is observable at about 1.5 µm away from the surfaces.

Understanding the interplay between structural transformation of cells and their response to mechanical challenges provides deeper mechanistic insight into mechanoregulation during EMT on all time scales. The large-scale structural changes during EMT are expected to cause substantial changes in cellular mechanics. Particularly, dissociation of cell-cell contacts representing a tension-generating boundary, as well as the degradation of cortical actin accompanied by stress fiber formation and an increased cell-substrate adhesion should have a strong impact on the cell's mechanical behavior. To quantify these time-dependent alterations we performed force indentation experiments (Fig. 2A) as well as tether pulling experiments (Fig. 2B) on living untreated and treated NMuMG cells using atomic force microscopy. We assume that the restoring force in response to indentation of the cells is predominantly generated by a constant isotropic tension *T* comprising the sum of cortical and overall tension *T*_0_ and a term describing the stretching or area dilatation of the bilayer (eq. 1). The associated area compressibility modulus *K_A_* of the plasma membrane is usually in the range of 0.1 N/m emphasizing the inextensibility of the lipid bilayer, while that of the underlying actin mesh is expected to be substantially smaller. Generally, bending and tension due to pre-stress prevails at low penetration depths, larger deformation resulting in stretching of the surface is governed by plasma membrane mechanics. Since the membrane is largely inextensible allowing only 2–3% of area dilatation, stretching of the bilayer leads in first order to a contribution described by a 2-D Hookean behavior that adds to membrane's overall tension (eq. 1). Therefore, the restoring force *F* generated by the isotropic tension *T* increases nonlinearly with indentation depth with a dominant contribution from area dilatation of the plasma-membrane at large strains. The tension *T_0_* is dominated by the cortical tension generated by the active actomyosin cortex, the attachment of the plasma membrane to the cytoskeleton, and adhesion due to cell-cell or cell-substrate contacts. The theoretical procedure to parameterize the cell in order to obtain force indentation curves to describe experimental data is given in the supporting information (Supplementary Text S1) following the publication of Discher and coworkers [28]. The shape of the cell prior to indentation is essentially a spherical cap described by the two topographical parameters *R_1_* and the contact angle *φ*.

In case of tether pulling experiments only tension within the membrane is addressed, whereas upon indentation the cellular membrane and the underlying actin cortex are deformed (Fig. 2A, left panel). Consequently, application of the two techniques enables us to address the contribution of both membrane tension *T_t_ and* cortical tension *T_C_* to the overall apical tension or pretension *T_0_* of the cells (eq. 6).
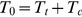
(6)Pretension or in-plane tension *T_0_* of the cortical shell including the cellular membrane and the actin cytoskeleton as well as the apparent area compressibility modulus *K_A_* ^′^ (Fig. 2A, right panel) were extracted from experimental force indentation curves by fitting the tension model to the data [28], [30], [43]. A detailed description of the procedure is given in the supplementary part of the manuscript. Notably the shape of the cells plays an important role to accurately determine the area compressibility modulus since the surface area is an important input parameter.

**Figure 3:**
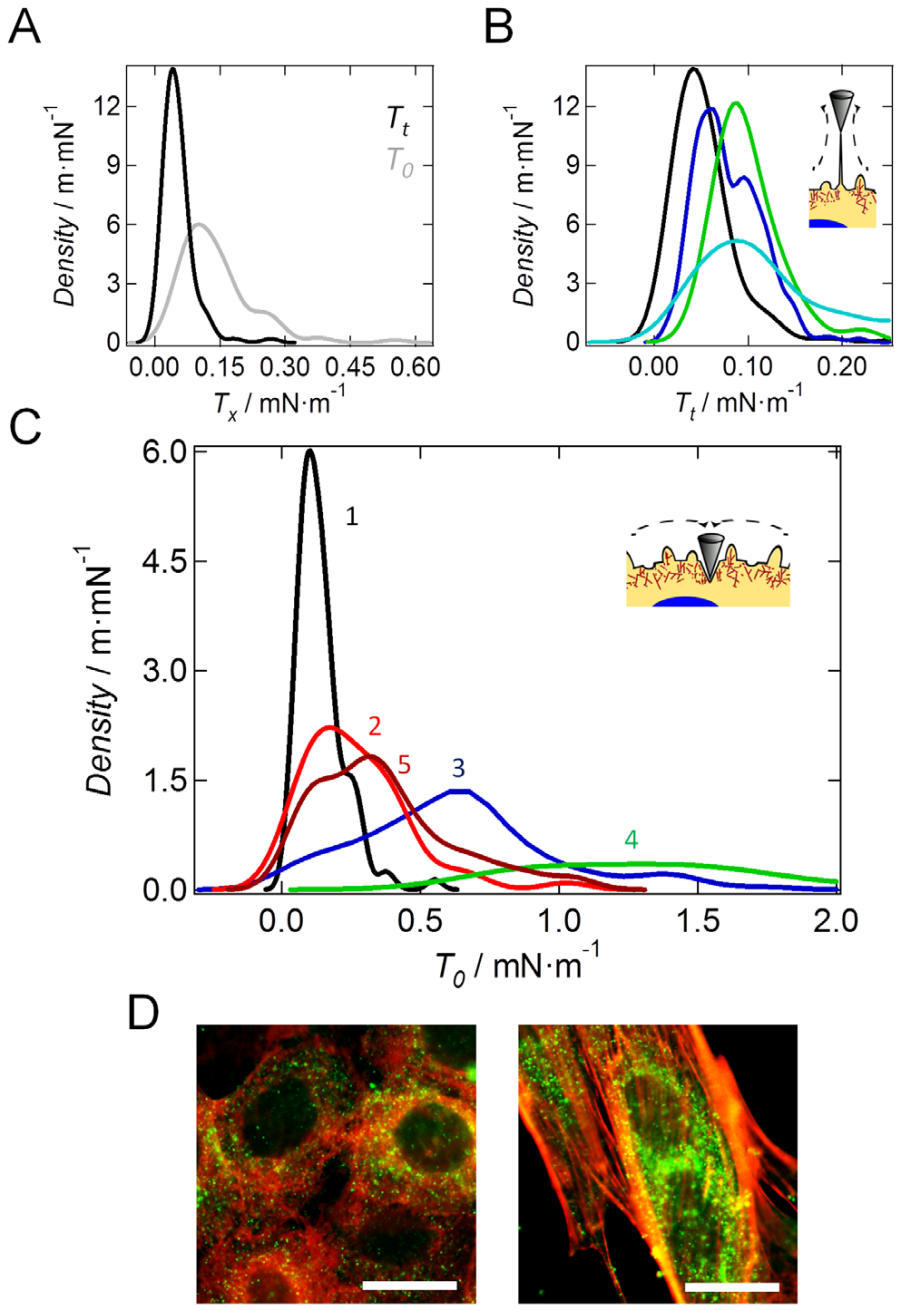
Mechanical properties of NMuMG cells under various conditions. (A) Comparison of tension values *T_x_* (kernel density functions), in which *T_x_* can be *T_0_* or *T_t_*, obtained from either indentation (grey, *n* = 108) or tether pulling (black, *n* = 109) experiments of untreated NMuMG cells. (B) Results from tether pulling experiments with untreated NMuMG cells (black, *n* = 109), NMuMG cells treated 48 h with TGF-β1 (blue, *n* = 274), single untreated NMuMG cells (green, *n* = 63) and fibroblasts (turquoise, *n* = 143). *n* depicts the number of tethers used for calculation. (C) Analysis of force indentation curves using a tension model [28], [30], [43]. Membrane tension (kernel density function) obtained from indentation experiments of untreated NMuMG cells (grey, labeled with number 1, *n* = 108), NMuMG cells treated 24 h with TGF-β1 (red, labeled with number 2, *n* = 52), NMuMG cells treated 48 h with TGF-β1 (blue, labeled with number 3, *n* = 95), single untreated NMuMG cells (green, labeled with number 4, *n* = 34) and NMuMG cells treated 48 h with TGF-β1 and 10 µg/ml cytochalasin D for 10 min (brown, labeled with number 5, *n* = 61) *n* depicts the number of curves used for calculation. In each experiment the velocity for cantilever approach and retraction during either indentation or tether pulling was 1 µm/s. For each category at least 4 cells were analyzed. (D) Fluorescence images of untreated NMuMG cells (left image) and NMuMG cells treated for 48 h with TGF-β1 (right image). Actin staining is shown in red, whereas the ERM protein moesin is stained in green. Scale bars: 20 µm.

Mainly a broadening of the distribution of *T_0_* including a slight shift of the maximum towards larger values was detectable 24 h after cytokine addition (transitional state, Fig. 3B). Prolongation of the incubation time up to 48 h (*mesenchymal-like* state) results in a strong increase of the tension values approaching those obtained for single untreated NMuMG cells (Fig. 3C). In contrast to confluent NMuMG cells bearing a cubical shape, solitary NMuMG cells show a more elongated morphology and an increased spreading behavior due to the absence of cell-cell contacts and a stronger cell-substrate adhesion including a high tendency for stress fiber formation (Fig. S6). Furthermore, the migratory behavior of cells organized in a confluent monolayer is much lower than that of single isolated NMuMG cells mirrored in lamellipodium formation in the latter case. Consequently, as solitary NMuMG cells bear nearly the same cellular morphology as NMuMG cells within the *mesenchymal-like* state, it is not surprising that also their mechanical behaviors are comparable. However, the broad distribution of the calculated pre-tension *T_0_*, still comprising values found for untreated cells, reflects a somewhat transitional state of the cells. To exclude that local effects during indentation determine the obtained results, colloidal probe indentation experiments were performed (Fig. S7) confirming that apical tension significantly rises from about 0.04 mN/m±0.01 mN/m (*n* = 10) before the transition to 0.11 mN/m±0.09 mN/m (*n* = 10) 48 h after EMT induction (Wilcoxon rank sum test, p-value<0.01). As stated above mainly structural changes during EMT are responsible for alterations of the mechanical properties of the cells. In order to evaluate the impact of the most relevant endogenous alterations taking place during the transition, we used a set of agents influencing distinct cellular process necessary for a proper proceeding of EMT (Fig. S8). Interestingly, inhibition of the translational elongation during protein synthesis by application of cycloheximide as well as administration of the Rho-kinase inhibitor Y-27632 does not alter membrane tension of the *mesenchymal-like* state. However, either pre-incubation of the cells with rapamycin, an inhibitor of cellular motility and *de novo* protein synthesis, or addition of blebbistatin lead to a significant decline of apical tension. Consequently, we assume that the increase in apical tension can be attributed to a higher acto-myosin contraction and the formation of actin stress fibers (Table 1).

**Table 1:** Tension values of NMuMG cells under various conditions obtained from AFM indentation measurements.

Treatment	*T_0_*/mN m^−1^
epithelial state	0.12±0.04 (*n* = 109)
*mesenchymal-like* state (TGF-β1)	0.61±0.01 (*n* = 274)
cycloheximide+TGF-β1	0.642±0.005 (*n* = 55)
rapamycin+TGF-β1	0.483±0.009 (*n* = 254)
blebbistatin+TGF-β1	0.570±0.007 (*n* = 235)
Y-27632+TGF-β1	0.710±0.003 (*n* = 153)
cytochalasin D+TGF-β1	0.288±0.005 (*n* = 100)

The assumption is supported by the fact that after incubation of *mesenchymal-like* NMuMG cells with the actin-depolymerizing agent cytochalasin D, a shift to lower tension values is found (Fig. 3C). This important result shows the strong influence of the cytoskeleton on apical tension during the transition. Interestingly, membrane tension remains unaffected after application of cytochalasin D. Besides stress fiber formation, upregulation and clustering of moesin at the ventral membrane side of NMuMG cells, which has already been described by Haynes *et al.* [22] and which can also be observed in our fluorescence micrographs (Fig. 3D) might have a strong impact on cellular mechanics by increasing the coupling strength between the membrane and the underlying actin composite. Similarly, Larson and coworkers [44] described the strong influence of ERM molecules on cortical mechanics in mouse oocytes during completion of meiosis and generation of cell polarity. Performing tether pulling experiments with concanavalin A coated cantilevers (Fig. 2B) [45] leads to a clear confinement of membrane tension from cortical tension as during these measurements only the cellular membrane is addressed (equation 6). Moreover, we corrected membrane tension *T_t_* for viscous contributions by using different cantilever retraction velocities (Fig. S9) according to Hochmuth *et al.* (1982) [46] and Krieg *et al.* (2008) [35] (eq. 5). By doing so, we calculated membrane tensions of 0.005 mN·m^−1^ and 0.052 mN·m^−1^ for NMuMG cells within either the epithelial or the *mesenchymal-like* state, respectively, as well as a viscosity coefficient *η* of 7.0·10^−7^ N·s·m^−1^ (Fig. S9). Comparable values for *η* have been found by Marcus and Hochmuth (2002) [47] and Sun *et al.* (2007) [48] for neutrophils and bovine aortic endothelial cells, respectively. Although both parameters *T_0_* and *T_t_* raise dramatically during EMT, the actin cortex highly dominates apical tension in either state. Whereas in case of NMuMG cells within the epithelial state, the contribution of membrane tension and cortical tension to the overall apical tension are 6% and 94%, respectively, their impact only slightly shifts to 8% and 92% after completion of EMT. Moreover, the calculated tension values from tether pulling experiments (Fig. 3B) show a bimodal distribution after transition (48 h incubation time) with a second peak at about 0.25 mN/m being in the same regime than tension values found for single NMuMG cells as well as for fibroblasts. Therefore, we assume that besides apical tension also membrane tension of cells that undergo EMT approaches that of single NMuMG cells.

### Viscoelastic alterations of NMuMG cells during EMT

**Figure 4:**
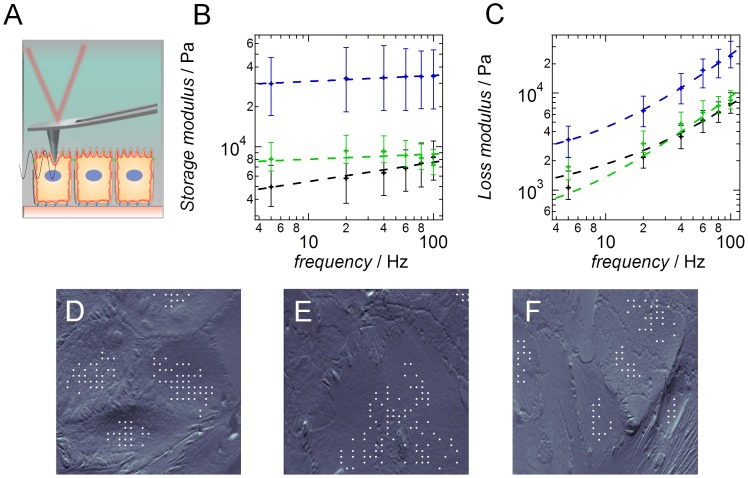
Viscoelastic properties of NMuMG cells during epithelial-to-mesenchymal transition. (A) Scheme of microrheological setup. Upon indentation of the sample, the AFM cantilever is excited sinusoidally and force curves are recorded within a predefined area. (B) Calculated storage modules of untreated NMuMG cells (black, *n* = 106), NMuMG cells during EMT (green, *n* = 90) and NMuMG cells within the final *mesenchymal-like* state (blue, *n* = 73). (C) Calculated loss modules. Color coding as in B. The dashed lines show the corresponding fits according to the power-law structural damping model [51]. *n* depicts the number of curves used for calculation. (D–F) AFM deflection images of living NMuMG cells during the transition. Untreated NMuMG cells (D), NMuMG cells treated 24 h with TGF-β1 (E) and NMuMG 48 h after administration of TGF-β1 (F). White points mark the positions of the recorded force curves on the cellular bodies used for analysis omitting the stiffer cell-cell borders.

The complex structure of cellular systems including the highly dynamic architecture of the cytoskeletal filaments underlines the necessity of suitable models for a more profound understanding of the cells' mechanical properties. Especially treating cells as purely elastic bodies is inadequate in most of the cases as they are known to exhibit viscoelastic behavior. Therefore, we recorded frequency-dependent rheological data according to a method introduced by Shroff and coworkers [49]. Upon indenting the cells, the AFM cantilever is excited sinusoidally at a given frequency (5 Hz to 100 Hz, Fig. 4A) and the obtained force curves were analyzed using the soft glassy rheology model. The model provides us with information about the elastic (storage modulus, *G′*) and viscous (loss modulus, *G″*) properties of the cells (Fig. 4B,C) [37]. Mainly, the frequency dependence of the elastic modules allows to infer, whether cells are solid-like or behave as a liquid thus displaying a more invasive potential. In case of a solid-like behavior the storage modulus G′ is generally higher than the loss modulus G″ within the recorded frequency range. A more liquid-like behavior provides the contrary result. The soft glassy rheology model treats the cytoskeleton as a network of individual structural elements, i.e. actin fibers, which are trapped in harmonic energy wells. Active motion, driven by the motor proteins, promotes hopping of the elements from one energy well to another. Most of the energy is restored by trapping the elements in a neighboring harmonic potential, while some of the energy is dissipated in the framework of heat. Upon administration of this model the calculated loss tangent *η* (*η* = *G*″/*G′*) represents mechanical friction coupled to the rate of crosslink-cycling, *G*′ relates to the number of acto-myosin bridges and the cortical tension, which promote the storage of energy, and *α* reflects the so called ‘noise temperature’ of the material. Applying this model to the data, it becomes apparent that the most prominent change in viscoelastic spectra is the substantial but only temporal increase in *α*, which is simply the slope of lg(*G*′) vs. lg(*ω*) shown in Fig. 4B [50], [51]. Soft glassy rheology (SGR) interprets *α*, the power law exponent, as the effective lattice temperature that similar to *k*_B_ *T* supplies energy for remodeling of the cytoskeleton thus facilitating hopping from energy well to energy well. In other words the power-law exponent *α* indicates how far the elements (actin cortex) are away from their equilibrium state. Although the energy wells are somewhat abstract with respect to cell biology and do not necessarily refer to spatially defined molecular entities they basically represents constraint to molecular motion. In case of untreated NMuMG cells both modules *G′* and *G″* rise with increasing frequency according to a weak power law (Fig. 4B,C). Interestingly, upon induction of the EMT, the storage modulus becomes largely independent of frequency, whereas the loss modulus doesn't change significantly (Fig. 4C). The loss modulus representing the amount of dissipated energy within the sample and therefore hinting at the viscous signature of the cells only increases after completion of the EMT (Fig. 4F blue and black curve, respectively). Moreover after finalization of the transition NMuMG cells become much stiffer (Fig. 4B). These results underscore that mainly elastic properties of NMuMG cells are altered during transition from the epithelial to the mesenchymal phenotype, most likely due to an increase in tension mediated by an altered cytoskeletal structure and the concomitant decrease in membrane area. These findings are also reflected in the parameters obtained by applying the power law structure damping model [51] (Table S1). During these measurements only force curves recorded on the center of the cellular bodies are considered as the cell borders are generally stiffer leading to a decrease in significance of the obtained results (Fig. 4D–F).

### Time-dependent delocalization and down-regulation of E-cadherin

**Figure 5:**
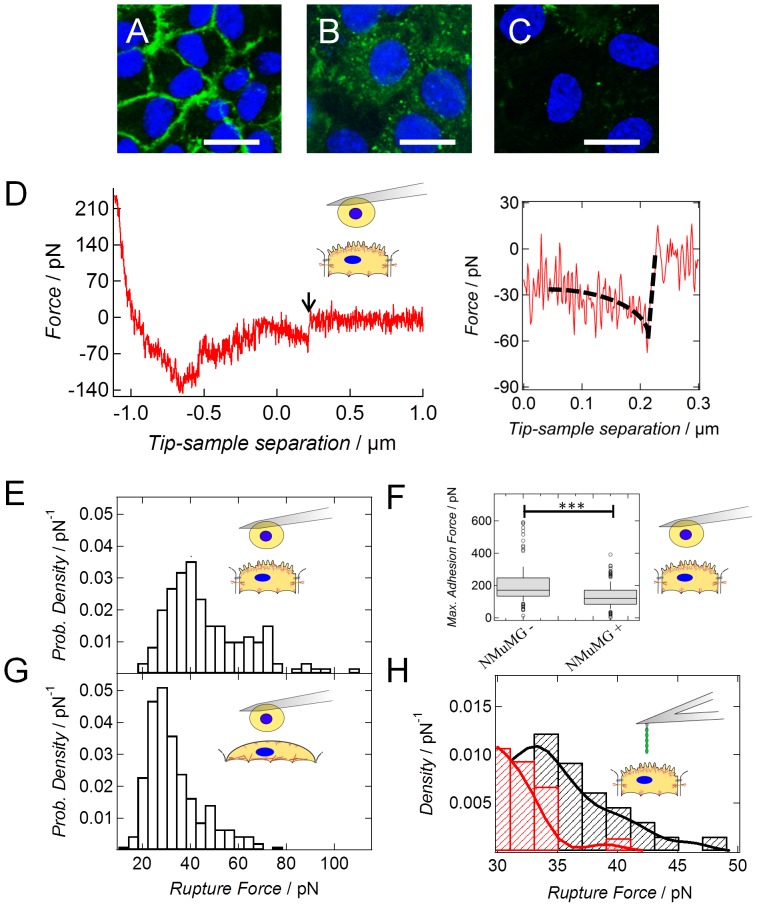
Alterations in E-cadherin expression and localization during EMT. (A) NMuMG cells in the epithelial state showing localization of E-cadherin at the cell-cell borders. (B) NMuMG cells incubated 48 h with the cytokine TGF-β1. Here a delocalization and increased intracellular uptake of E-cadherin is observable. (C) NMuMG cells incubated 10 d with the cytokine TGF-β1. After this long incubation time, expression of E-cadherin is completely down-regulated. Staining of the nucleus was carried out with DAPI (blue). A monoclonal Alexa Fluor488-conjugated IgG2a antibody was used to stain E-cadherin (green). Scale bar: 20 µm. (D) Typical retraction curve from single cell force spectroscopy measurements showing the interaction of two untreated NMuMG cells. The arrow marks a characteristic rupture event attributed to a specific homomeric cadherin interaction. Inlay displays the rupture event in higher magnification. A contact time of 1 sec between the cells was chosen. (E/G) Histograms of single rupture forces obtained from single cell force spectroscopy measurements using a contact time of 1 sec in either case (D). Force curves were recorded with an effective loading rate (force per time) of *v_r_* = 2.3 nN/s for epithelial cells and of *v_r_* = 0.7 nN/s for cells within the *mesenchymal-like* state, respectively. Strength of homomeric cadherin binding of either two untreated NMuMG cells (E, *n* = 149) or two NMuMG cells treated 8–10 d with TGF-β1 (G, *n* = 230) were investigated. (F) Maximum adhesion force of either two epithelial cells (NMuMG −, *n* = 175) or two *mesenchymal-like* cells (NMuMG +, *n* = 285) obtained from the minima in the retraction curves (Fig. 5D).*** P<7.7·10^−16^ (Wilcoxon rank sum test). Both categories are significantly different from each other. (H) Kernel density function of single rupture forces. A single E-cadherin molecule was brought into contact with an epithelial cell (black, *n* = 327) or with an epithelial cell pre-incubated for 1 h under physiological conditions with an E-cadherin antibody (red, *n* = 373) to abolish specific interactions. Contact time between the single molecules and the cells was 1 sec under both conditions. The effective loading rate *v_r_* was 0.2 nN/s.

During EMT intercellular junctions are strongly impaired as observed for NMuMG cells (Fig. 5A–C). The significant decline in apical tension (p-value<0.01) after administration of EDTA emphasizes the importance of cell-cell contacts for cellular mechanics (Fig. S10). Calcium depletion also interferes with cytoskeleton integrity, which also reduced the measured cortical tension. Therefore, a more profound understanding of the temporal alterations of cell-cell adhesion during EMT including a quantification of the exerted forces between the cells even down to the single-molecule level is of fundamental importance. Especially, the concerted and time-dependent down-regulation of the adherens junction protein E-cadherin is essential for epithelial-to-mesenchymal transition [52]. Whereas the amount of E-cadherin remains unchanged within the first 2–3 days after cytokine addition, the localization of the protein is completely altered. The strong membrane displacement of E-cadherin implicates that binding to the actin cytoskeleton is abrogated (Fig. 5B). Interestingly, a complete down-regulation is observed only 8 d after cytokine addition, as described previously [53] (Fig. 5C). These findings were confirmed with the help of a fluorescence intensity analysis. Upon summing up the intensity values of the E-cadherin fluorescence images (Fig. 5A–C), we calculated a value of 3759 a.u. for NMuMG cells in the epithelial state, 4610 a.u. for NMuMG cells 48 h after EMT induction and a value of 976 a.u. for NMuMG cells within the mesenchymal state. The fluorescence images were recorded using the same time (300 ms) and intensity for illumination. As cadherins are known for mediating a physical coupling between neighboring cells [13], a strong influence on cellular mechanics is expected as already reviewed by Cavey and Lecuit in 2009 [54]. Therefore, we investigated the overall interaction force of two opposing cells, either two untreated NMuMG cells (epithelial state) or two NMuMG cells incubated with TGF-β1 for at least 8 d (*mesenchymal-like* state), as well as their interaction on the single molecule level via single cell force spectroscopy (SCFS), and single molecule force spectroscopy. Figure 5D shows a typical force retraction curve of two epithelial cells being brought into contact. The maximum adhesion force decreases (Fig. 5F) significantly from about 171 pN to 120 pN after cytokine stimulation (p<0.001). More specific than the overall adhesion force is the occurrence of single rupture events documenting individual unbinding events of E-cadherin dimers (Fig. 5D in-lay). To evaluate whether the single rupture events prior to EMT can indeed be attributed to homomeric E-cadherin interactions, *single* E-cadherin molecules covalently bound to an AFM cantilever via a thiol linker are used for adhesion mapping of the cellular surface. The obtained histograms taken from force volume data of several cells show a characteristic maximum for untreated NMuMG cells at about 35 pN being in good accordance with SCFS data (*vide infra*). This force maximum vanishes upon addition of an E-cadherin antibody (red curve, Fig. 5H).

Analyzing the recorded force distance curves obtained from cell-cell adhesion measurements, we detect a bimodal distribution of the single rupture forces hinting at the detachment of more than one E-cadherin molecule during cantilever retraction. However as inferred from single molecule experiments using single E-cadherin constructs attached to the cantilever *and* the underlying substrate, we also found a bimodal distribution (Fig. S10). It is therefore conceivable that instead of a detachment of multiple E-cadherin proteins a multistate unbinding of single molecules takes place as already inferred from Leckband and coworkers [55]. Therefore, we compared only the first peaks at low forces within the histograms of untreated and treated NMuMG cells. By doing so, we clearly see a decline of the unbinding force from 40 pN to 28 pN during EMT (Fig. 5E,G). Differences in the detected rupture forces by either single-cell or single-molecule force measurements can be attributed to the absence of an intracellular domain in the latter case contributing to the strength of cadherin-cadherin bonds [56] as well as dissimilar loading rates used during the experiments. However, similar to the findings of Evans and Calderwood [57], we revealed only a small influence of the effective loading rate on rupture force of the homomeric E-cadherin bond (Fig. S11B), which is too weak to explain the shift of the interaction forces of single unbinding events from 40 pN down to 28 pN during EMT. The weak dependence of the rupture force on loading rate is a good example for a persistent cellular adhesion binding in contrast to transient connectors as VCAM-1 [49]. The number of successful adhesion events during each set of experiments only slightly varies, especially in case of the cell-cell interaction experiments (Table S2).

In summary, these results support the hypothesis and expectation that mainly down-regulation of E-cadherin is responsible for the weakening of intercellular interactions during epithelial-to-mesenchymal transition.

## Discussion

In this study, we investigated the mechanistic link between structural transformation of the epithelial cell line NMuMG during epithelial-to-mesenchymal transition and the corresponding mechanical response to external probing. Therefore, we performed different modes of force spectroscopy to gain insight into the mechanistic response of NMuMG cells during different stages of EMT. Especially the combination of indentation and tether pulling experiments is highly suitable to extract the influence of membrane tension *and* cortical tension on the overall stress present within the apical part of the cell [43]. Indenting isolated apical membrane sheets with the AFM and comparing their elastic response to that of adherent epithelial cells, we already depicted that the cellular membrane dominates the mechanical properties of epithelial cells [29], [58]. Generally the apical pretension is regulated by the buffering membrane reservoir (vesicles, caveolae e.g.), and generated by adhesion of the cortical actin to the membrane mainly mediated by ERM proteins, the osmotic pressure within the cell, and the strength of cellular junctions [11], [15], [16]. Since a high number of structural alterations takes place during EMT including formation of actin stress fibers [59], loss of cell-cell contacts [60], as well as changes in cell-matrix adhesion [61], these effects are likely candidates to substantially alter cellular mechanics and at the same time challenge membrane integrity. Particularly, the importance of cell-cell contacts for the maintenance of apical tension becomes evident upon addition of the chelator agent EDTA, which leads to a significant decline of the apical tension determined from AFM indentation experiments (Fig. S11), since the pre-stress exerted by the adherens junctions is suddenly released. Using single-cell force spectroscopy we were now able to quantify the weakening of the overall cellular adhesion upon cytokine stimulation. These results are in good agreement with findings from literature describing a decline of the maximum unbinding force required for separation of two HK2 cells after their transition [62]. Loss of the presence of cell-cell contacts is partly mediated by delocalization and degradation of the adherens junction protein E-cadherin counterbalanced by an increased expression of N-cadherin [63]. Therefore, we determined a shift of the mean single molecule rupture forces from 40 pN between two epithelial cells to 28 pN for two cells in the *mesenchymal-like* state, which we attribute to down-regulation and internalization of E-cadherin [64], as well as to a weakening of the cadherin-catenin complex being highly important for strengthening of cellular adhesion [65]. The remaining interaction forces are due to unspecific van der Waals and electrostatic interactions and not to the unbinding of cadherins. This is supported by the fact that no second peak within the force distribution is detectable, which is normally highly characteristic for multistate unbinding of cadherins as stated by Leckband and coworkers [49]. Using single-molecule force spectroscopy we could verify that single rupture events determined from cell-cell measurements are in fact E-cadherin-E-cadherin interactions. The subsequent decline in the detected tension by loss of cell-cell contacts is most likely balanced by a highly dynamic regulation of excess membrane area, most probably by a loss of membrane reservoirs such as caveolae [18] and a concomitant rise in cell-substrate adhesion. The latter assumption is supported by the fact that the overall occupied area of NMuMG cells increases by approximately 20% as already stated by Lamouille and Derynck [23]. The loss of membrane area stored in folds and protrusions during adjustment of cell adhesion is supported by a roughness analysis of AFM deflection images revealing a smoothening of the membrane during EMT. Loss of membrane area during EMT has already been depicted by us via ECIS (electric cell-substrate impedance) measurements detecting a decrease in membrane capacity [66].

Surprisingly, although the cellular structure alters completely during the transition (Fig. 1A,B) [66], only a minor change in apical tension for NMuMG cells was detectable within the first 24 h. Since regulation of tension is important for many processes like exo- and endocytosis, as well as cell motility, growth or division [67], [68], [69], [70], we conclude that the cell compensates for the structural changes during EMT within the first 24 h of cytokine stimulation by mechanically adapting to the new morphology. However, as inferred from AFM indentation experiments longer incubation times lead to a strong increase of the calculated values approaching those determined for solitary NMuMG cells. The increase in apical tension includes also a rise in pure membrane tension as confirmed by tether pulling experiments providing an independent means to measure site-specific membrane tension without the influence of the actin cytoskeleton. Interestingly, we depicted that - similar to the overall apical tension - pure membrane tension of NMuMG cells incubated 48 h with TGF-ß1 is in the same regime than lateral membrane tension found for single NMuMG cells (about 0.2 mN/m), as well as for fibroblasts showing mesenchymal properties.

However, administration of cytochalasin D to NMuMG cells in the *mesenchymal-like* state leads to a decline in *T_0_* to values comparable to those obtained during the transitional phase of EMT. Therefore it is conceivable that reorganization of the actin cytoskeleton highly influences the mechanical properties of the cells during the transition. Mainly formation of stable stress fibers and the concomitant changes in cell-substrate adhesion [61] reflected in a higher spreading area as well as the strong attachment of the membrane to the underlying composite via an increased expression of moesin as deduced by our fluorescence intensity analysis might contribute to this result. The impact of adhesion on membrane tension has already been investigated by us upon compressing giant liposomes using a modified AFM setup [71]. According to these calculations an increase in contact radius of 0.1 µm leads to a rise in membrane tension of about 0.07 mN·m^−1^. Considering the increase in apical tension of a factor of 6 (epithelial state: 0.10 mN·m^−1^, *mesenchymal-like state*: 0.64 mN·m^−1^) during EMT, it is conceivable that the alteration in spreading area highly contributes to the increased tension. Furthermore, upregulation of ERM proteins affects the mechanical properties of cells and is involved in important cellular processes as cell polarity or meiosis [44]. In combination with findings from Haynes and coworkers who reported that an increased moesin expression highly promotes EMT by contributing to actin reorganization, cell-substrate adhesion and an increased cellular migration [22], we conclude that characteristic changes in membrane tension are necessary for cellular transdifferentiation processes like the EMT. This assumption is supported by the fact that moesin is highly essential for a proper regulation of mitosis [72], which shows parallels with EMT concerning alterations in cellular mechanics and the generation of physical forces [73]. Furthermore, micro-rheological data acquired during different stages of EMT induced by administration of TGF-ß1 reflects a stabilization of the cytoskeleton from epithelial to *mesenchymal-like* phenotype. According to the power law structural damping model cells become mechanically quiescent displaying a more tensed cortex in the *mesenchymal-like* state than in the epithelial state reflected in decreasing *α* values during the transition.

**Figure 6:**
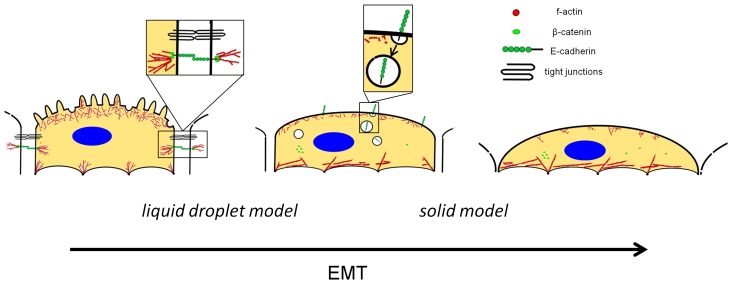
Scheme illustrating the structural alterations during epithelial-to-mesenchymal transition. Within the epithelial state, membrane tension is mainly dominated by cell-cell interactions (tight and adherens junctions), the adhesion of the cytoskeleton to the membrane via ERM proteins and the buffering membrane reservoir (e.g. folds and microvilli). Upon transformation, actin stress fibers are formed and cell-substrate adhesion increases accompanied by a loss of membrane area. Structural changes (e.g. loss of cortical actin and intercellular junctions) include a reduction in surface area leading to homeostasis of membrane tension (*cell in the middle*). Destabilization of the cadherin complex [76], followed by E-cadherin delocalization and internalization take place during the transition. Within these early time regimes, the cells can be treated as liquid droplets surrounded by a membrane with static tension. However, within the final *mesenchymal-like* state membrane protrusions are stretched out allowing the cell to increase its spreading area on the surface. Due to this enforced cell-substrate adhesion in conjunction with an increased stress fiber formation, the mechanical response of *mesenchymal-like* cells has to be described as a viscoelastic solid.

Generally, a larger value of *α* corresponds to a ‘hot’ state in which dynamic structural rearrangements occur to a larger extent, while a smaller value of α refers to a ‘cold’ state where the cells assumes a mechanically frozen state with high stiffness and low ‘noise temperature’, i.e. reduced amount of molecular agitation. This increase in cellular mechanics can be attributed to the occurring reorganizational processes including loss of cell-cell contacts and rearrangement of the actin cytoskeleton. Especially the higher expression and clustering of moesin and α-SMA, as well as an higher acto-myosin contraction accompanied by stress fiber formation might lead to the observed weakening of the power law dependency as already inferred by treating HASM cells with the contractile agonist histamine [74]. Within the epithelial state, NMuMG cells can be regarded as liquid droplets whose mechanical properties are dominated solely by the surrounding cellular membrane. During EMT the overall structure of the cells changes completely resulting in a mechanical behavior that resembles that of much stiffer cells like chondrocytes [75] (Fig. 6) supporting the idea that the influence of the cortical actin increases by stress fiber formation and up-regulation and clustering of moesin. Within the *mesenchymal-like* state the impact of the cellular membrane on the mechanical properties determined by indentation experiments is smaller and is most likely replaced by the elastic properties of the cytoskeleton including newly generated stress fibers.

The switch from a viscoelastic behavior to a more elastic one has already been shown by treating mesenchymal stem cells, as well as 3T3 fibroblasts with 4T1 breast tumor cell conditioned media finally promoting an increased cellular migration [77]. Similarly, it has been reported that a higher membrane tension results in an increased motility as this leads to a more organized lamellipodium and hence a higher migration rate [78]. In the same line are the results from Diz-Munoz who claimed that membrane-to-cortex attachment regulates cellular migration at least in vivo [79]. Consequently, it is conceivable that the migratory behavior during EMT increases; a well-known characteristic of this transition [23]. This is also supported by our finding that pretreatment of NMuMG cells with the mTOR signaling pathway inhibitor rapamycin [80] 1 h before TGF-β1 administration leads to a decrease in membrane tension (Table 1 and Fig. S8). As rapamycin is known to decrease cellular dynamics [27] and the motility of TGF-β1 treated NMuMG cells [23], we assume that membrane tension and cellular motility are directly liaised to each other. Preincubation with blebbistatin [25] leads also to smaller tension values indicating that actomyosin contraction participates in an increased membrane tension and is indeed necessary for a complete EMT as described by Haynes and coworkers [22]. Furthermore, neither stress fiber formation nore *de novo* protein synthesis are involved as indicated by administration of the Rho-kinase inhibitor Y-27632 [81] and the protein inhibitor cycloheximide [24], respectively (Table S2).

Tension regulation during epithelial-to-mesenchymal transition plays an important role in protecting cells from damages due to severe remodeling of the cytoskeleton and seems to be a prerequisite for an increased cellular motility. New formation of stress fibers at the expense of cortical actin accompanied by elongation of cells poses enormous mechanical stress, but seems to be relevant for a proper proceeding of this phenotype switching. However, due to their liquid-crystalline nature membranes are incapable to bear large strains, i.e. area dilatation is limited to merely few percent of the initial area. Therefore, it is of great importance to prevent membrane damage by regulating membrane tension during the whole process. We have shown that EMT is controlled by a highly responsive feedback mechanism to track and correct for changes of tension. Consequently, we assume that membrane tension can be considered as an important set point markedly influencing cellular morphology, mechanics and motility during such an intricate process as EMT (Fig. 6).

## Supporting Information

Text S1 **Mathematical description of the mechanical model used to model the data.**
(PDF)

Table S1 **Viscoelastic properties of NMuMG cells during EMT.** Fitting parameters obtained from microrheology data after application of the power law structural damping model [13].
(PDF)

Table S2 **Number and percentage of analyzed curves with at least one single rupture event obtained from AFM force retraction curves under various conditions as well as the number of cells which was taken into account.** The absolute number of the recorded curves is also given. Interestingly, the percentage of single adhesive events during contact of two epithelial (Epith-Epith) is similar to that during interaction of two cells within the *mesenchymal-like* state (Mes-Mes). This result mirrors that the detected decline in adhesion force during EMT (Fig. 5 E/G) is not due to a lower number of single rupture events. The fact that the percentage of interactions between single E-cadherin molecules and epithelial NMuMG cells (E-Cad-Epith) is slightly lower than that after addition of the E-cadherin antibody (E-Cad-Epith+Ab) can be elucidated with an unspecific interaction of the E-cadherin-bound antibody with either the cellular surface or the cantilever.
(PDF)

Figure S1 **Typical force indentations as a function of area compressibility modulus** ***K*** **_A_ assuming a pre-tension of** ***T_0_*** **= 0.3 mN m^−1^, an initial cell radius** ***R_0_*** **= 35 µm and a half opening angle of the conical indenter of α = 17.5°** ***K*** **_A_: (orange) 0.05 N m^−1^, (red) 0.1 N m^−1^, (black) 0.2 N m^−1^, (green) 0.3 N m^−1^, (blue) 0.4 N m^−1^.**
(PDF)

Figure S2 **Force curves with varying** ***T_0_*** **values assuming** ***K_A_*** **= 0.2 N m^−1^, an initial cell radius** ***R_0_*** **= 35 µm and a half opening angle of the conical indenter of α = 17.5°.** ***T_0_*** **: (orange) 0.1 mN m^−1^, (red) 0.2 mN m^−1^, (black) 0.3 mN m^−1^, (green) 0.4 mN m^−1^, (blue) 0.5 mN m^−1^.**
(PDF)

Figure S3 **Influence of cell radius** ***R_0_*** **on force indentation curves (** ***R_0_*** **: (red) 20 µm, (black) 25 µm, (blue) 30 µm, (green) 35 µm, (light blue) 40 µm, (pink) 45 µm, (orange) 50 µm, (grey) 45 µm).** The following parameters were assumed: Wetting angle prior to indentation *φ* = 20°, half opening angle of the conical indenter α = 17.5°, area compressibility modulus *K_A_* = 0.2 N/m, and tension *T*_0_ = 0.1 N/m.
(PDF)

Figure S4 **Influence of cellular shape (wetting angle) on force indentation curves.** An initial cell radius *R_0_* = 35 µm (note that *R_1_* = *R_0_* sin(*φ*)) and a half opening angle of the conical indenter of α = 17.5° are used for computation of force indentation curves. A typical area compressibility modulus of *K_A_* = 0.2 N/m and a pre-stress of *T*_0_ = 0.1 N/m were assumed for all data. *φ*: (red) 16°, (yellow) 18°, (black) 20°, (green) 25°, (pink) 30°, (orange) 40°, (light blue) 50°, (blue) 60°.
(PDF)

Figure S5 **AFM height images of NMuMG cells within the epithelial state (A), NMuMG cells 24 h after EMT induction (B) and NMuMG cells within the final** ***mesenchymal-like*** **state (C).** The white square highlights the region chosen for surface roughness analysis. Cells have been fixed using glutardialdehyde prior AFM closed-loop contact imaging. MLCT cantilevers were conducted and a scan rate of 0.2 Hz was chosen. Setpoint and gains were adjusted during imaging.
(PDF)

Figure S6 **Colloidal probe indentation experiments. Exemplarily chosen force-distance curve recorded by indenting an untreated NMuMG cell (red) and a NMuMG cell treated 48 h with the cytokine TGF-β1 (blue) with a colloidal probe.**
(PDF)

Figure S7 **Cellular structure and morphology of solitary NMuMG cells.** (A) AFM deflection image of single NMuMG cells showing an elongated morphology Instead of strong cell-cell contacts, these solitary cells strongly adhere to the underlying substrate mirrored in a high number of stress fibers whose presence is visualized in AFM height images (B).Prior imaging, cells have been stained with 2.5% glutardialdehyd solution diluted in 1× PBS^−^ (15 min incubation time). A MLCT cantilever with a nominal spring constant of 0.01 N/m and a scan rate of 0.2 Hz were used.
(PDF)

Figure S8 **Mechanical properties of NMuMG cells in the** ***mesenchymal-like*** **state after pretreatment with various drugs.** (A) Membrane tension *T_0_* (kernel density function) obtained from force-indentation experiments of NMuMG cells treated 48 h with TGF-β1 (blue, *n* = 95), NMuMG cells preincubated with 100 nM rapamycin for 1 h before TGF-β1 addition (grey, *n* = 254), NMuMG cells preincubated with a 18 µM cycloheximide solution for 1 h before TGF-β1 addition (blue, *n* = 55), NMuMG cells preincubated with 10 µM blebbistatin for 1 h before TGF-β1 addition (black, *n* = 234) and NMuMG cells preincubated with 15 µM Y-27632 for 1 h before TGF-β1 addition (green, *n* = 153). In all of the cases TGF-β1 incubation was carried out for 48 h. *n* depicts the number of curves used for calculation. (B) Fluorescence images of TGF-β1 treated NMuMG cells preincubated for 1 h with various agents as indicated. Scale bars: 25 µm.
(PDF)

Figure S9 **Tether forces obtained from AFM tether pulling experiments at different velocities of 5, 10 and 20 µm/s using ConcanavalinA coated cantilevers and epithelial NMuMG cells.** According to equation 5 we are able to correct membrane tension *T_t_* for viscous contributions. The slope of the fit (black dashed line) enables us to calculate the viscosity coefficient *η*, whereas the intersection of the fit with the ordinate directly provides us with the tether force without viscous contributions.
(PDF)

Figure S10 **Membrane tension of NMuMG cells under various conditions.** Tension values *T_0_* of untreated NMuMG cells (*n* = 108) and NMuMG cells incubated with 2 mM EDTA diluted in 1× PBS^−^ (*n* = 28; *** p-value<0.01; Wilcoxon rank sum test). EDTA was added to the sample 10 min before the measurement was started. Values are obtained from force indentation experiments with a pyramidal indenter according to our tension model.
(PDF)

Figure S11 **Interaction of single isolated E-cadherin molecules.** (A) Histograms monitoring either rupture forces of E-cadherin-E-cadherin bonds (black) or of E-cadherin-E-cadherin bonds after addition of the chelator agent EDTA (grey) obtained from AFM retraction curves. The proteins are attached via a thiol linker either to a gold-coated cantilever or to a gold coated substrate. In both cases the molecules stand perpendicular to the surface and show homophilic interactions upon cantilever approach. EDTA was added in a concentration of 2 mM diluted in 1× PBS^−^. (B) Rupture force of the homomeric E-cadherin interaction as a function of the effective loading rate *r*_f_. Data from cell-cell (red point), single molecule-cell (green point) and single molecule-single molecule experiments (black points) are included into this plot. The grey dashed line shows the corresponding fit to the data points. Interestingly, over a range of five orders of magnitude the detachment force differs only 10 pN in maximum.
(PDF)
