# Supplementary material for: Tension Monitoring during Epithelial-to-Mesenchymal Transition Links the Switch of Phenotype to Expression of Moesin and Cadherins in NMuMG Cells
Source: PLoS One. 2013 Dec 5;8(12):e80068. doi: 10.1371/journal.pone.0080068 (PMC3855076; doi:10.1371/journal.pone.0080068)
Supplement: Text S1 — Mathematical description of the mechanical model used to model the data. (PDF) [file pone.0080068.s001.pdf]

Supplementary Text

1 Mechanical model of cell indentation by atomic force microscopy

The following paragraphs briefly summarize how to extract tension T_0 and area compressibility K_A from indentation experiments based on the theory published by Sen *et al.* (*Biophys. J.*, 2005, **89**, 3203-3213.).

1.1 Parameterization of the cell's shape

In this section we outline how to assess the cellular shape during indentation with a conical indenter. Adhesion of cells to the surface leads to shapes that can best be described as capped spheres with contact angles around $\phi_0 = 60\,^\circ$. We consider the cell as an isotropic elastic shell that produces a restoring force in response to indentation with a conical indenter originating from two sources, linear elasticity due to area dilatation at large strains and and pre-stress (constant tension) stored in the membrane and actin cortex. Tension of the cortex/membrane composite is mainly generated by active elements (actomyosin), adhesion to the surface and interaction of the plasma membrane with the cytoskeleton mediated by, for instance ERM proteins. Bending, however, plays a minor role and is therefore neglected in the following theoretical description. The tension of the plasma membrane can be written as:

$$T = T_0 + K_a \frac{A - A_0}{A_0}, \tag{1}$$

in which T_0 comprises cortical tension T_c of the actomyosin cortex, and T_t the membrane tension including elements of adhesion to the cytoskeleton and in–plane tension of the membrane.

$$T_0 = T_c + T_t, \tag{2}$$

K_A is the area compressibility modulus of the plasmamembrane, $\Delta A = A_{cl} - A_0$ is the difference between the actual area A_{cl} after compression and the initial area prior to compression A_0. Static equilibrium can be expressed by the Young-Laplace equation, which describes the pressure difference across the fluid interface, which is the pressure difference between interior and exterior of the cell as a function of surface tension T and mean curvature $H = \frac{1}{2}\left(\frac{1}{R_m} + \frac{1}{R_\phi}\right)$. R_m and R_ϕ denote the meridional and circumferential radii of curvature at any point in the membrane.

$$\Delta P = 2TH \tag{3}$$

Assuming a constant curvature of the free membrane justified by a constant hydrostatic pressure difference and a constant isotropic tension, we arrive at

$$2H = \frac{du}{dr} + \frac{u}{r}, \tag{4}$$

where $u = \sin\beta$. β denotes the angle between the surface normal to the vertical axis. Equation (4) is solved for the following boundary conditions (see scheme 1 for definition of parameters):

$$\beta = -\Theta, r = r_1 \tag{5}$$
$$\beta = \phi, r = R_1 \tag{6}$$

The shape function u can be written as:

$$u = C_1 r + \frac{C_2}{r}, \tag{7}$$

with

$$C_1 = \frac{R_1 \sin(\phi) + r_1 \sin(\Theta)}{R_1^2 - r_1^2} \tag{8}$$
$$C_2 = -C_1 r_1^2 - r_1 \sin(\Theta) \tag{9}$$

1.2 Force acting on the cantilever

Force equilibrium at the base provides a mean to access force as a function of ϕ and r_1:

$$\pi R_1^2 \Delta P = F + \int_0^{2\pi R_1} T \sin(\phi)\, \mathrm{d}l \tag{10}$$
$$\pi R_1^2 \Delta P = F + 2\pi R_1 \sin(\phi) T \tag{11}$$
$$\pi R_1^2 T 2H = F + 2\pi R_1 \sin(\phi) T \tag{12}$$
$$\tag{13}$$

with

$$2H = \frac{du}{dr} + \frac{u}{r} = 2C_1, \tag{14}$$

and eventually

$$F = 2\pi T (R_1^2 C_1 - R_1 \sin(\phi)) \tag{15}$$

1.3 Computation of the cellular shape as a function of indentation depth

The task is now to determine ϕ and r_1, which constitute essentially the shape of the indented cell. Therefore, we assume that the volume of the cell is preserved during the indentation process. The volume can be computed from the shape as a function of indentation depth:

$$V = \int_{r_1}^{R_1} \pi r^2 \frac{(C_1 r - \frac{C_2}{r})}{\sqrt{1 - (C_1 r + \frac{C_2}{r})^2}}\, \mathrm{d}r - \frac{\pi r_1^3}{3\tan(\Theta)} \tag{16}$$

and be set equal to the volume of the cell prior to indentation (shape of a spherical cap):

$$V = (3R_0 - h)\frac{\pi h^2}{3}, \tag{17}$$

with h the height of the cell according to scheme 1 (note that $R_0 = R_1/\sin(\phi_0)$):

$$h = R_0 - \sqrt{R_0^2 - h}. \tag{18}$$

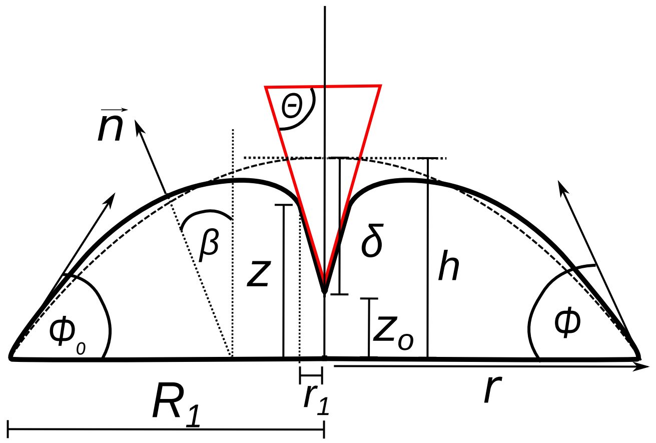

Scheme 1: An adherent cell represented by a spherical cap (dotted line) subject to indentation using a conical indenter (continuous line). The indenter is shown in red. Illustration of mechanical parameters used in the mechanical tension model.

The tension T, however, is not constant but increases with area dilatation. Assuming a 2-D Hookean solid we arrive at:

$$T = T_0 + K_a\alpha, \tag{19}$$

with

$$\alpha = \frac{\Delta A}{A_0} \tag{20}$$

α denotes the area dilatation with A_0, the area prior to indentation The actual area A_{cl} can be readily obtained by solving the integral

$$A_{cl} = \int_{r_1}^{R_1} \frac{2\pi r}{\sqrt{1-(C_1 r + \frac{C_2}{r})^2}}\,\mathrm{d}r + \frac{\pi r_1^2}{\sin(\Theta)} \tag{21}$$

The two non-linear equations (15,16) are solved numerically for $[r_1, \phi]$ using the function `fsolve` from `MATLAB`^{TM} which finds a root of a system of nonlinear equations employing the Levenberg-Marquardt algorithm. The z-position of the AFM–tip at $r = r_1$ is calculated from

$$dz = dr\tan(\beta), \tag{22}$$

$$dz = \frac{u}{\sqrt{1-u^2}}. \tag{23}$$

Integration provides the height $z(r_1)$:

$$z(r_1) = \int_0^{r_1} \frac{u}{\sqrt{1-u^2}}\,\mathrm{d}r \tag{24}$$

that allows us to determine the indentation depth δ:

$$\begin{aligned}
z_0 &= z(r_1) - r_1 \tan(\Theta); & (25)\\
\delta &= h - z_0, & (26)\\
\delta &= h - \int_0^{r_1} \frac{u}{\sqrt{1-u^2}}\,\mathrm{d}r + r_1 \tan(\Theta), & (27)
\end{aligned}$$

The procedure allows to obtain $F(\delta)$ curves by computing the shape of the cell parameterized by r_1 and ϕ.
