# Supplementary material for: Tension Monitoring during Epithelial-to-Mesenchymal Transition Links the Switch of Phenotype to Expression of Moesin and Cadherins in NMuMG Cells
Source: PLoS One. 2013 Dec 5;8(12):e80068. doi: 10.1371/journal.pone.0080068 (PMC3855076; doi:10.1371/journal.pone.0080068)
Supplement: Table S1 — Viscoelastic properties of NMuMG cells during EMT. Fitting parameters obtained from microrheology data after application of the power law structural damping model [13]. (PDF) [file pone.0080068.s002.pdf]

Table S1

	G_0 / Pa	α	μ / Pa s
epithelial state	3996	0.16	12
transitional state	6178	0.03	12
mesenchymal-like state	12439	0.06	23
