# Supplementary material for: Tension Monitoring during Epithelial-to-Mesenchymal Transition Links the Switch of Phenotype to Expression of Moesin and Cadherins in NMuMG Cells
Source: PLoS One. 2013 Dec 5;8(12):e80068. doi: 10.1371/journal.pone.0080068 (PMC3855076; doi:10.1371/journal.pone.0080068)
Supplement: Table S2 — Number and percentage of analyzed curves with at least one single rupture event obtained from AFM force retraction curves under various conditions as well as the number of cells which was taken into account. The absolute number of the recorded curves is also given. Interestingly, the percentage of single adhesive events during contact of two epithelial (Epith-Epith) is similar to that during interaction of two cells within the mesenchymal-like state (Mes-Mes). This result mirrors that the detected decline in adhesion force during EMT (Fig. 5 E/G) is not due to a lower number of single rupture events. The fact that the percentage of interactions between single E-cadherin molecules and epithelial NMuMG cells (E-Cad-Epith) is slightly lower than that after addition of the E-cadherin antibody (E-Cad-Epith+Ab) can be elucidated with an unspecific interaction of the E-cadherin-bound antibody with either the cellular surface or the cantilever. (PDF) [file pone.0080068.s003.pdf]

Table S2

	Epith-Epith	Mes-Mes	E-Cad-Epith	E-Cad-Epith + Ab
**No. of curves used for analysis**	91 of 175 (52%)	148 of 285 (52%)	212 of 615 (31%)	297 of 671 (44%)
**No. of cells**	15	9	6	6
