# Supplementary figures and images for: Tension Monitoring during Epithelial-to-Mesenchymal Transition Links the Switch of Phenotype to Expression of Moesin and Cadherins in NMuMG Cells
Source: PLoS One. 2013 Dec 5;8(12):e80068. doi: 10.1371/journal.pone.0080068 (PMC3855076; doi:10.1371/journal.pone.0080068)

**Figure S1**

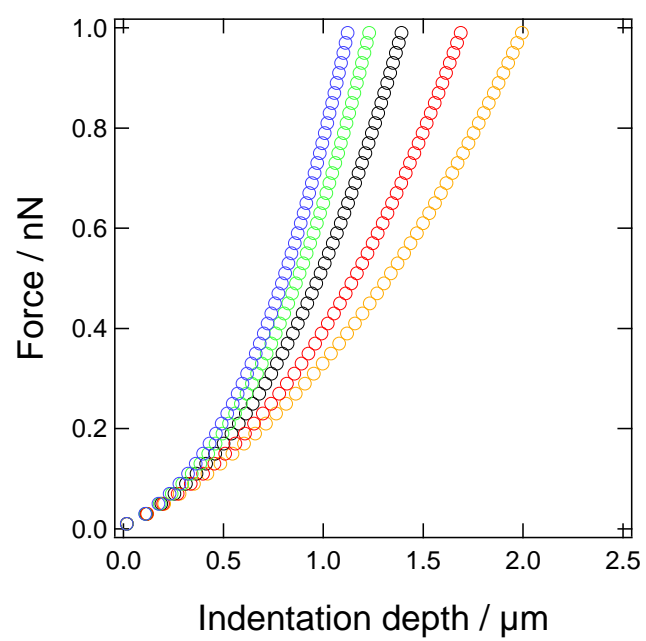

Supplement: Figure S1 — Typical force indentations as a function of area compressibility modulus K A assuming a pre-tension of T0 = 0.3 mN m−1, an initial cell radius R0 = 35 µm and a half opening angle of the conical indenter of α = 17.5° K A: (orange) 0.05 N m−1, (red) 0.1 N m−1, (black) 0.2 N m−1, (green) 0.3 N m−1, (blue) 0.4 N m−1. (PDF) [file pone.0080068.s004.pdf]

**Figure S2**

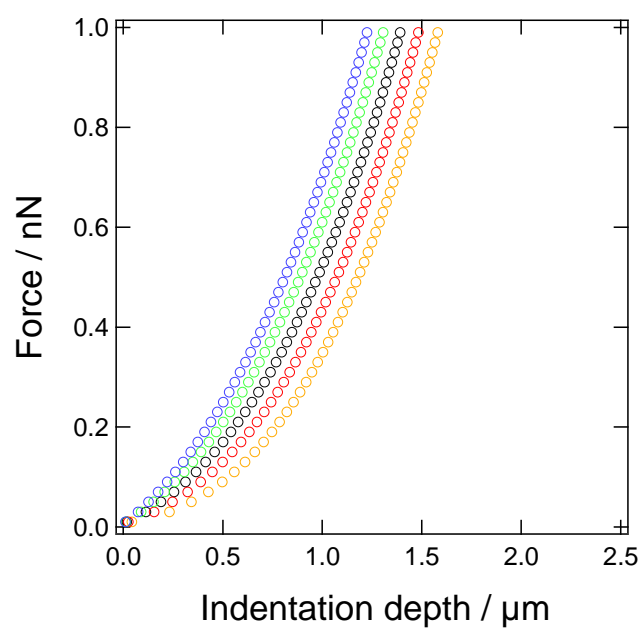

Supplement: Figure S2 — Force curves with varying T0 values assuming KA = 0.2 N m−1, an initial cell radius R0 = 35 µm and a half opening angle of the conical indenter of α = 17.5°. T0 : (orange) 0.1 mN m−1, (red) 0.2 mN m−1, (black) 0.3 mN m−1, (green) 0.4 mN m−1, (blue) 0.5 mN m−1. (PDF) [file pone.0080068.s005.pdf]

**Figure S3**

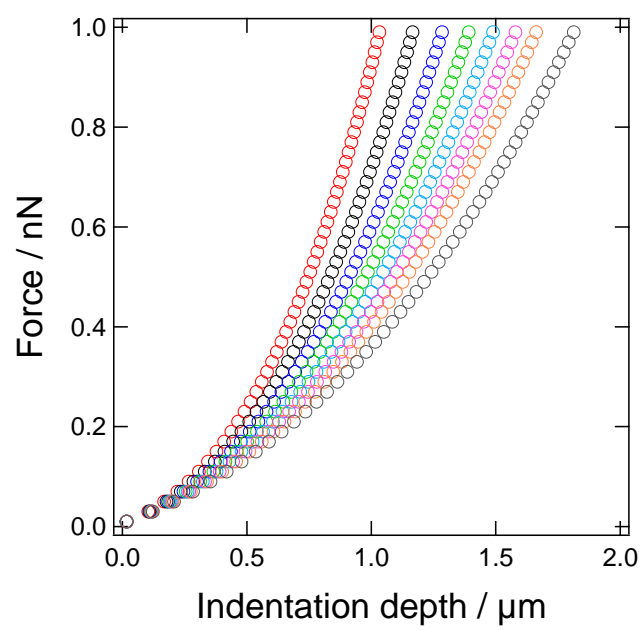

Supplement: Figure S3 — Influence of cell radius R0 on force indentation curves ( R0 : (red) 20 µm, (black) 25 µm, (blue) 30 µm, (green) 35 µm, (light blue) 40 µm, (pink) 45 µm, (orange) 50 µm, (grey) 45 µm). The following parameters were assumed: Wetting angle prior to indentation φ = 20°, half opening angle of the conical indenter α = 17.5°, area compressibility modulus KA = 0.2 N/m, and tension T 0 = 0.1 N/m. (PDF) [file pone.0080068.s006.pdf]

**Figure S4**

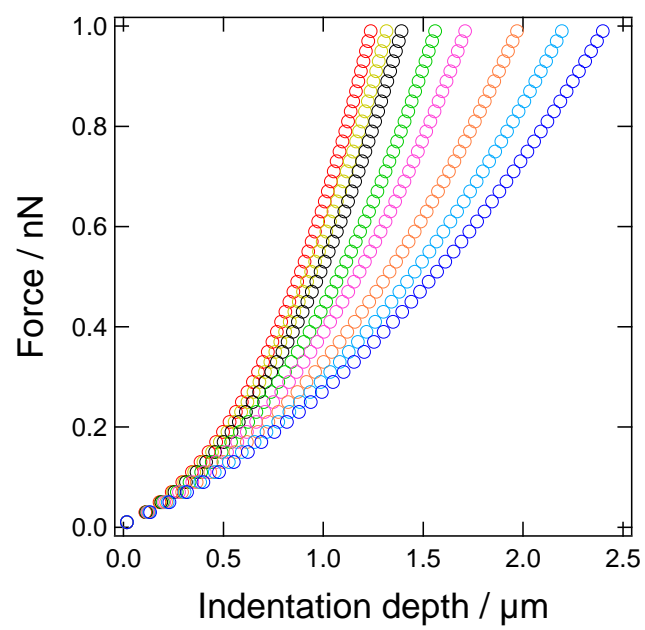

Supplement: Figure S4 — Influence of cellular shape (wetting angle) on force indentation curves. An initial cell radius R0 = 35 µm (note that R1 = R0 sin(φ)) and a half opening angle of the conical indenter of α = 17.5° are used for computation of force indentation curves. A typical area compressibility modulus of KA = 0.2 N/m and a pre-stress of T 0 = 0.1 N/m were assumed for all data. φ: (red) 16°, (yellow) 18°, (black) 20°, (green) 25°, (pink) 30°, (orange) 40°, (light blue) 50°, (blue) 60°. (PDF) [file pone.0080068.s007.pdf]

**Figure S5**

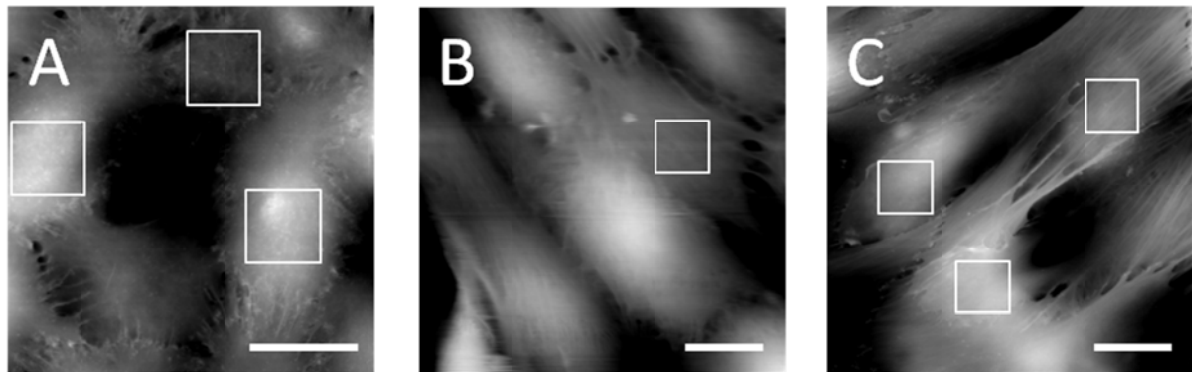

Supplement: Figure S5 — AFM height images of NMuMG cells within the epithelial state (A), NMuMG cells 24 h after EMT induction (B) and NMuMG cells within the final mesenchymal-like state (C). The white square highlights the region chosen for surface roughness analysis. Cells have been fixed using glutardialdehyde prior AFM closed-loop contact imaging. MLCT cantilevers were conducted and a scan rate of 0.2 Hz was chosen. Setpoint and gains were adjusted during imaging. (PDF) [file pone.0080068.s008.pdf]

**Figure S6**

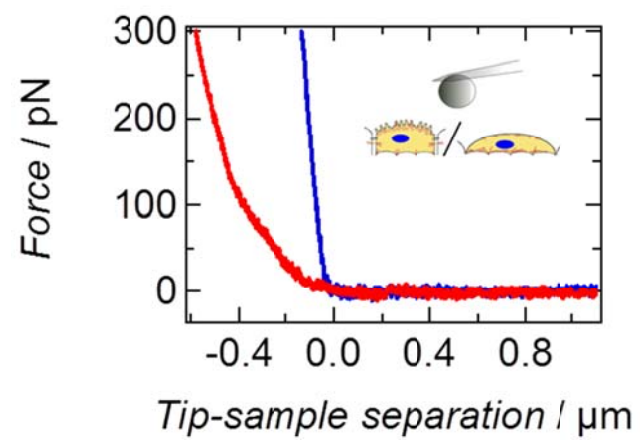

Supplement: Figure S6 — Colloidal probe indentation experiments. Exemplarily chosen force-distance curve recorded by indenting an untreated NMuMG cell (red) and a NMuMG cell treated 48 h with the cytokine TGF-β1 (blue) with a colloidal probe. (PDF) [file pone.0080068.s009.pdf]

**Figure S7**

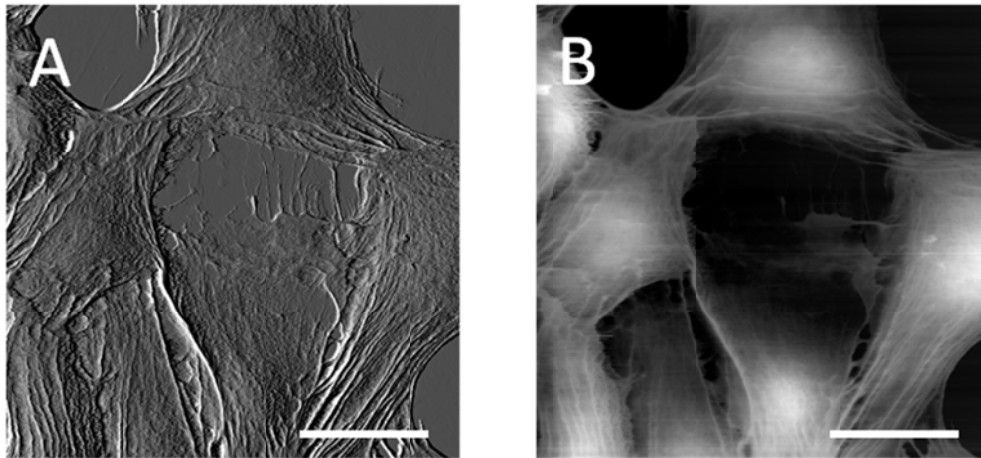

Supplement: Figure S7 — Cellular structure and morphology of solitary NMuMG cells. (A) AFM deflection image of single NMuMG cells showing an elongated morphology Instead of strong cell-cell contacts, these solitary cells strongly adhere to the underlying substrate mirrored in a high number of stress fibers whose presence is visualized in AFM height images (B).Prior imaging, cells have been stained with 2.5% glutardialdehyd solution diluted in 1× PBS− (15 min incubation time). A MLCT cantilever with a nominal spring constant of 0.01 N/m and a scan rate of 0.2 Hz were used. (PDF) [file pone.0080068.s010.pdf]

Figure S8

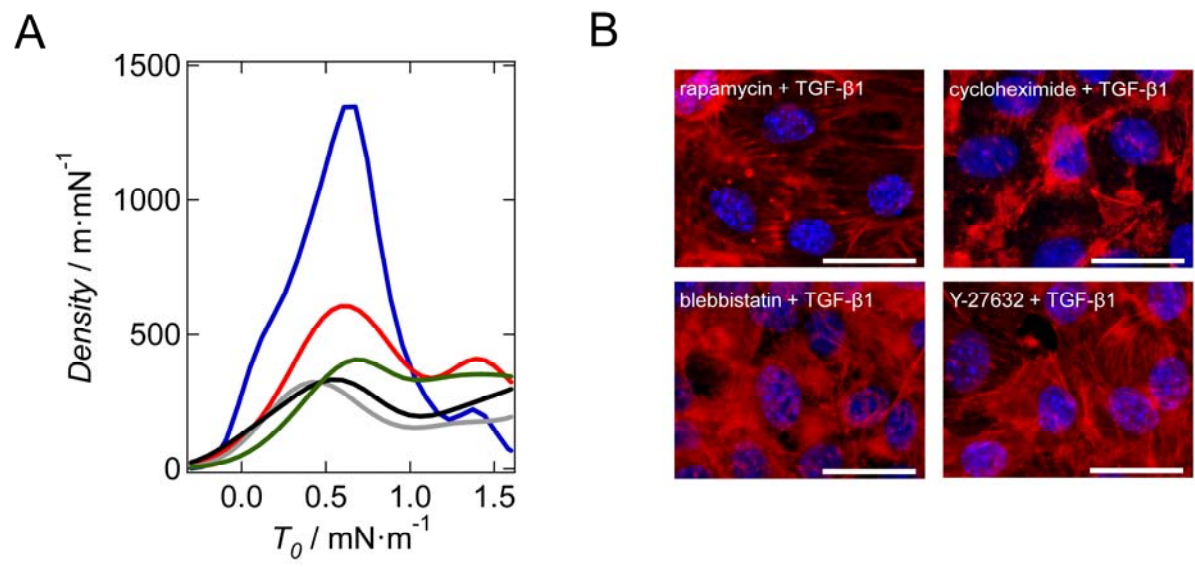

Supplement: Figure S8 — Mechanical properties of NMuMG cells in the mesenchymal-like state after pretreatment with various drugs. (A) Membrane tension T0 (kernel density function) obtained from force-indentation experiments of NMuMG cells treated 48 h with TGF-β1 (blue, n = 95), NMuMG cells preincubated with 100 nM rapamycin for 1 h before TGF-β1 addition (grey, n = 254), NMuMG cells preincubated with a 18 µM cycloheximide solution for 1 h before TGF-β1 addition (blue, n = 55), NMuMG cells preincubated with 10 µM blebbistatin for 1 h before TGF-β1 addition (black, n = 234) and NMuMG cells preincubated with 15 µM Y-27632 for 1 h before TGF-β1 addition (green, n = 153). In all of the cases TGF-β1 incubation was carried out for 48 h. n depicts the number of curves used for calculation. (B) Fluorescence images of TGF-β1 treated NMuMG cells preincubated for 1 h with various agents as indicated. Scale bars: 25 µm. (PDF) [file pone.0080068.s011.pdf]

**Figure S9**

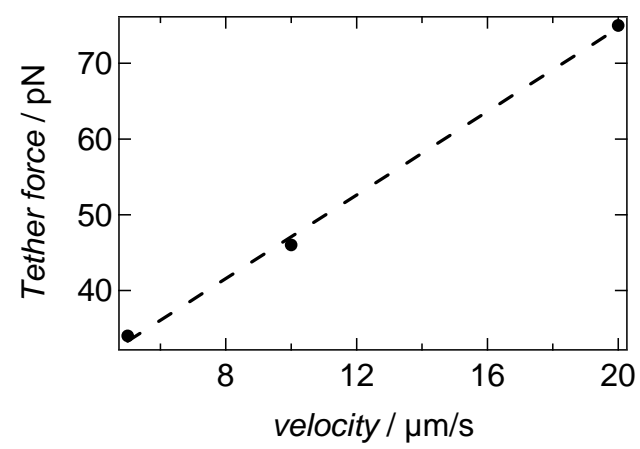

Supplement: Figure S9 — Tether forces obtained from AFM tether pulling experiments at different velocities of 5, 10 and 20 µm/s using ConcanavalinA coated cantilevers and epithelial NMuMG cells. According to equation 5 we are able to correct membrane tension Tt for viscous contributions. The slope of the fit (black dashed line) enables us to calculate the viscosity coefficient η, whereas the intersection of the fit with the ordinate directly provides us with the tether force without viscous contributions. (PDF) [file pone.0080068.s012.pdf]

**Figure S10**

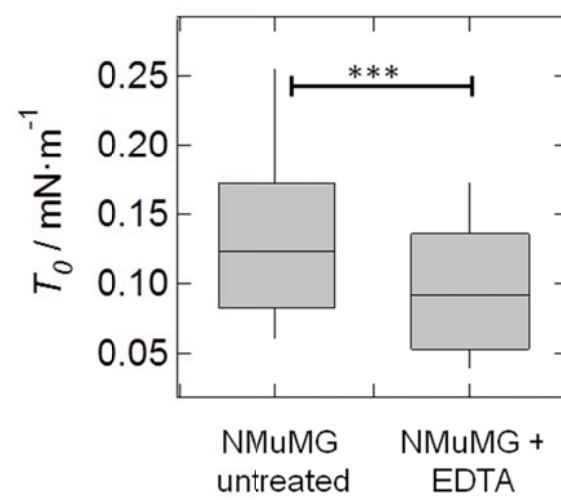

Supplement: Figure S10 — Membrane tension of NMuMG cells under various conditions. Tension values T0 of untreated NMuMG cells (n = 108) and NMuMG cells incubated with 2 mM EDTA diluted in 1× PBS− (n = 28; *** p-value<0.01; Wilcoxon rank sum test). EDTA was added to the sample 10 min before the measurement was started. Values are obtained from force indentation experiments with a pyramidal indenter according to our tension model. (PDF) [file pone.0080068.s013.pdf]

**Figure S11**

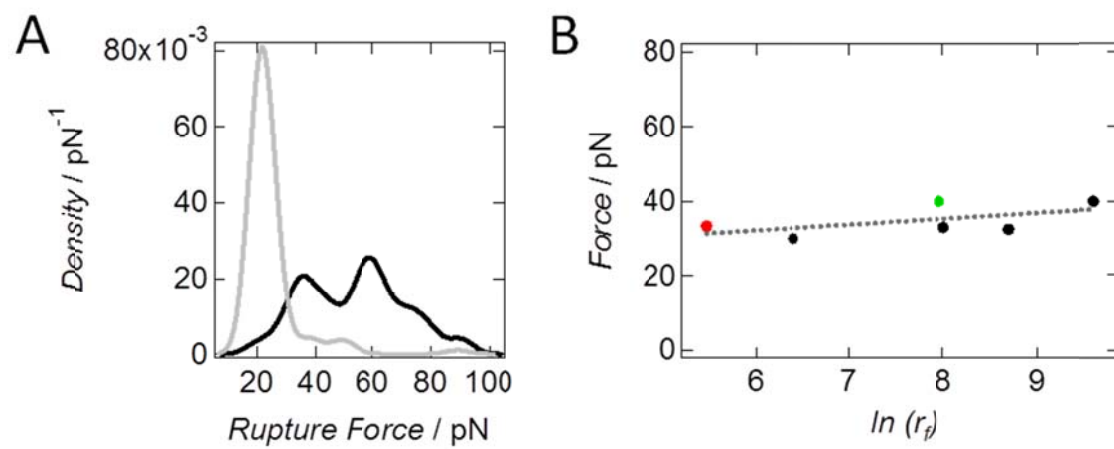

Supplement: Figure S11 — Interaction of single isolated E-cadherin molecules. (A) Histograms monitoring either rupture forces of E-cadherin-E-cadherin bonds (black) or of E-cadherin-E-cadherin bonds after addition of the chelator agent EDTA (grey) obtained from AFM retraction curves. The proteins are attached via a thiol linker either to a gold-coated cantilever or to a gold coated substrate. In both cases the molecules stand perpendicular to the surface and show homophilic interactions upon cantilever approach. EDTA was added in a concentration of 2 mM diluted in 1× PBS−. (B) Rupture force of the homomeric E-cadherin interaction as a function of the effective loading rate r f. Data from cell-cell (red point), single molecule-cell (green point) and single molecule-single molecule experiments (black points) are included into this plot. The grey dashed line shows the corresponding fit to the data points. Interestingly, over a range of five orders of magnitude the detachment force differs only 10 pN in maximum. (PDF) [file pone.0080068.s014.pdf]
